# Active immunization in patients transplanted for hepatitis B virus related liver diseases: A prospective study

**DOI:** 10.1371/journal.pone.0188190

**Published:** 2017-11-16

**Authors:** Anli Yang, Zhiyong Guo, Qingqi Ren, Linwei Wu, Yi Ma, Anbin Hu, Dongping Wang, Haidan Ye, Xiaofeng Zhu, Weiqiang Ju, Xiaoshun He

**Affiliations:** 1 Organ Transplant Center, The First Affiliated Hospital of Sun Yat-sen University, Guangzhou, China; 2 Guangdong Provincial Key Laboratory of Organ Donation and Transplant Immunology, The First Affiliated Hospital of Sun Yat-sen University, Guangzhou, China; 3 Guangdong Provincial International Cooperation Base of Science and Technology (Organ Transplantation), The First Affiliated Hospital of Sun Yat-sen University, Guangzhou, China; Yonsei University College of Medicine, REPUBLIC OF KOREA

## Abstract

**Introduction:**

Prophylactic administration of hepatitis B immunoglobulin (HBIG) and nucleos(t)ide analogues (NAs) is the standard treatment for controlling hepatitis B virus (HBV) recurrence after liver transplantation (LT). Since lifelong use of HBIG is expensive and inconvenient and the antibodies level in anti-hepatitis B surface (HBs) is not sustainable and stable, an alternative strategy is to produce anti-HBs antibodies by active immunization. Our present study aimed to prospectively investigate the efficacy and safety of procedural HBV vaccination in transplanted patients.

**Methods:**

Recipients who had undergone LT for hepatitis B related liver diseases more than one year before, with no evidence of HBV recurrence or rejection and normal liver function were enrolled. All subjects received the hepatitis B vaccine (40 μg) by intramuscular injection at months 0, 1, 2, 6 and 12 after enrollment with continuous administration of NAs. The liver function and anti-HBs titers were measured before each vaccination and HBIG (400U) was administrated intramuscularly when anti-HBs titer was lower than 30 IU/L during the course. The results of routine blood tests, liver function, concentration of immunosuppressant, and HBV-DNA copies were monitored during the research. After completion of the vaccination procedure, recipients were regarded as responders if their anti-HBs greater than 30 IU/L were maintained for up to six months without using HBIG and vaccine.

**Results:**

Twenty-seven patients were enrolled in this study and the average anti-HBs titer before vaccination was 19.86±14.80 IU/L. The average anti-HBs titer of the nine responders at the end of the follow-up was 57.14±22.75 IU/L, giving an overall response rate of 33.3% (9/27). There were no reports of reactivation of HBV, rejection, severe anaphylaxis or other adverse events. Responders and non-responders showed their significant difference in anti-HBs titers after the fourth vaccination (P<0.01). Moreover, the majority of non-responders (11/18, 63.64%) had high LY/EO rates (lymphocyte number/eosinophil number>15) while most responders (8/9, 88.89%) had low LY/EO rates at the beginning of vaccination (P = 0.019).

**Conclusions:**

Active immunization is an effective, cost-saving, and safe method for the prevention of HBV reactivation in patients transplanted for hepatitis B virus related liver diseases. The LY/EO rate may be a valuable indicator in selecting potential recipients for vaccination.

## Introduction

Hepatitis B virus (HBV), discovered half a century ago, is one of the most serious global infectious diseases. Worldwide, over 2 billion people have been exposed to HBV infections and about 3–5% of them become chronically infected, which causes liver cirrhosis and/or hepatocellular carcinoma [1, 2]. China has one of the world’s highest rates of chronic HBV carriers, as high as 10–20%, and up to 90% of liver transplantation (LT) is performed for patients with HBV-related liver diseases [3, 4]. Therefore, prophylactic management of post-transplant hepatitis B recurrence is an important issue for improving long-term survival, especially in China.

In the era without prophylaxis, over 70% of transplanted patients suffered from HBV recurrence, which used to make HBV-related liver diseases a relative contraindication for LT [5]. Thanks to the invention and application of anti-hepatitis B immunoglobulin (HBIG) and nucleos(t)ide analogues (NAs), outcomes of the aforementioned patients have improved markedly. Currently, the standard treatment for controlling hepatitis B recurrence after LT is combining HBIG with NAs, of which the safety and efficacy is undoubted [6, 7]. However, long-term administration of HBIG is associated with several unresolved issues, including limited availability, inconvenience and extremely high cost.

To reduce the medical and financial burden from HBIG, several centers have evaluated a combination of low-dose HBIG and NAs, including fixed low-dose and on-demand low-dose HBIG protocols, and monoprophylaxis with high genetic barrier NAs. Among these, active immunization against HBV has become a potential alternative, which seems attractive in terms of both reduction in cost and safety of recipients. However, the efficacy of the hepatitis B vaccine on transplant recipients is controversial due to the varieties in inoculation procedures and subject selection.

In this report, we prospectively studied the efficacy and safety of procedural HBV vaccination in patients transplanted for HBV related liver diseases. Additionally, we paid attention to characteristics that could discriminate between vaccine-responsive and non-responsive patients.

## Methods

### Subject selection

From September 2013 to August 2014, patients who had been transplanted for HBV-related liver diseases for more than one year and had no evidence of HBV recurrence or severe episodes of rejection were recruited at our outpatient clinic. They were receiving a combined prevention regimen using NAs and HBIG and their liver function was normal under stable low-level immunosuppression therapy at the time of enrollment. Additionally, all participants signed informed consent forms after fully understanding this trial.

We excluded patients who had ever suffered from surgical complications in the biliary tract or vessels, were ever diagnosed with another serious organ dysfunction, used to be hypersensitive to any ingredient of the hepatitis B vaccine, or presented any sign of tumor recurrence. None had coinfection with human immunodeficiency virus, hepatitis delta virus or hepatitis C virus.

None of the transplant donors were from a vulnerable population and all donors or next of kin provided written informed consent that was freely given. All organ donations and transplantations were approved by the Institutional Review Board of the First Affiliated Hospital of Sun Yat-sen University, under the guidelines of the National Health and Family Planning Commission, and the current regulations of the Chinese Government.

### Study protocol

**Fig 1:**
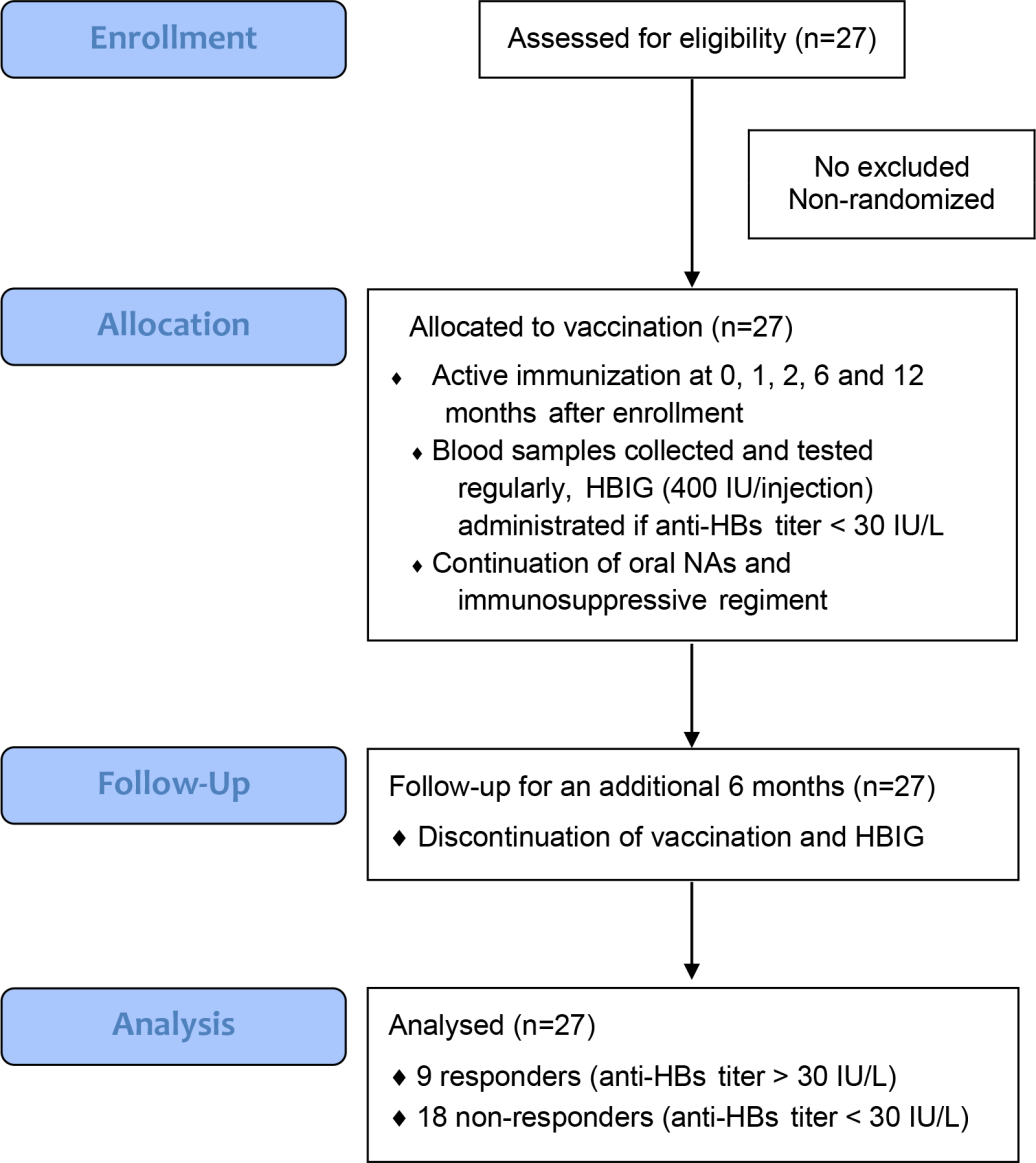
Schematic depiction of our study protocol.

A recombinant hepatitis B surface antigen (HBsAg) vaccine (Engerix-B, GSK) was officially approved for clinical use and commercially available in China. Schematic depiction of our study protocol is shown in Fig 1. All subjects received double-doses of the vaccine (40 μg) by intramuscular injection into deltoid muscle at 0, 1, 2, 6 and 12 months after enrollment. Blood samples were collected for laboratory tests on the day prior to inoculation. If the serum anti-HBs titer was lower than 30 IU/L, the participant would receive intramuscular injection of HBIG (400 IU) at least 2 weeks after the most recent inoculation in order to maintain a stable baseline level of anti-HBs titer. Oral NAs and immunosuppressive regiments stayed unchanged throughout the course.

After completion of the yearlong vaccination protocol, patients were followed for an additional six months, without continuation of vaccination and HBIG administration. At the end of our follow-up, patients were regarded as “responders” if their serum anti-HBs titer was higher than 30 IU/L; otherwise, they were classified as “non-responders”. Responders would use booster vaccination for maintaining anti-HBs titer, while non-responders were resumed on HBIG administration.

The study protocol was approved by the Ethics Committee of the First Affiliated Hospital of Sun Yat-sen University (No. 2013102) and was conducted in accordance with the Declaration of Helsinki principles. We enrolled our first subject in September 2013 after ethical approval, though registration on the Chinese Clinical Trial Registry was completed in December 2013. Therefore, three patients were enrolled before the completion of registration, but all of them received vaccinations in accordance with our study protocol. The registration number on http://www.chictr.org.cn/ is ChiCTR-ONC-13003942.

### Virological assays

Serum HBV markers including anti-HBs were detected quantitatively with a chemiluminescence immunoassay using an ARCHITECT i2000 (Abbott Diagnostics, USA) automated immunoassay analyzer. HBV DNA was detected with a real-time quantitative PCR diagnostic kit for quantification of hepatitis B virus DNA (Da an Gene Co., Ltd. of Sun Yat-sen University, China) using an ABI 7500 PCR instrument (Applied Biosystems, USA). The detection limit was 10^3^–10^7^ copies/ml.

### Statistical analysis

Normally or near normally distributed variables were reported as means with standard deviations (SD) and were compared using Student’s t-tests when applicable. Non-normally distributed continuous data were reported as medians with ranges and were compared using Mann–Whitney U-tests. Categorical variables were presented as numbers with percentages and compared using the χ2-test and Fisher’s exact test for fewer than five events. A p value of less than 0.05 was inferred as statistically significant. SPSS 13.0 (SPSS Inc., Chicago, IL, USA) was used for all statistical analyses.

## Results

### Characteristics of patients

Twenty-seven men were enrolled in the current study and their indications for LT were HBV related liver diseases, including fulminant hepatic failure (B-FHF) in four patients, decompensated cirrhosis (BLC) in eleven patients, hepatocellular carcinoma (HCC) in three patients and hepatocellular carcinoma with cirrhosis (HCC+BLC) in nine patients. At the time of vaccination, the median age of patients was 50 years (range 27–67 years), and a median of 790 days (range 390–4894 days) had elapsed since LT. The demographic, clinical and virological features of these patients are shown in Table 1.

**Table 1:** Demographic, clinical and virological characteristics of individual patients.

Case	Blood Type	Age	Etiology	Hepatitis B history (years)	MELD	Pre-LT			At the start of vaccination				Anti-HBs titer (IU/L)		
						HBsAg	Anti-HBs	HBV-DNA	Interval from LT (days)	BMI	Immuno- suppression	NAs	Before vaccine	End of vaccine	Last f/u
1	O	55	HCC+BLC	23	10	+*	-*	2.28*10^4^	514	27.68	TC+SR	ETV	53.76	29.78	26.58
2	O	40	BLC	4	19	0.02	0	2.6*10^3^	1607	23.51	SR	ETV	1.04	52.38	72.77
3	A	48	B-FHF	1	NA	+	-	NA	1441	29.27	TC	ADV	31.11	26.19	24.13
4	B	43	BLC	23	3	250	0	6.07*10^5^	686	21.22	TC+MMF	ADV	45.84	57.13	50.19
5	A	53	HCC+BLC	23	8	250	1.52	NA	563	21.80	TC+MMF	ETV	4.87	17.18	16.64
6	A	26	B-FHF	3	23	0.21	29.3	8.64*10^2^	482	26.30	TC+MMF	ETV	7.70	4.98	9.05
7	O	53	HCC+BLC	23	24	+	-	9.75*10^6^	3218	21.94	TC	LAM	19.87	73.04	53.95
8	A	34	BLC	13	14	0.39	0	NA	1750	24.22	TC	LAM	48.40	13.56	7.81
9	O	59	BLC	13	21	1.07	0	NA	2756	19.10	TC+MMF	LAM	30.96	46.31	23.05
10	A	64	HCC+BLC	1	4	1.23	0	<100	921	27.22	TC+MMF	ADV	4.89	1.89	2.69
11	O	37	BLC	14	24	+	-	<100	4894	21.97	CyA	LAM	10.63	10.6	13.17
12	O	46	HCC+BLC	23	6	+	-	3.38*10^3^	608	27.36	SR	ETV	15.38	45.48	53.16
13	O	48	HCC+BLC	6	22	18.21	0	NA	726	29.41	TC+MMF	LAM	26.08	33.48	44.79
14	A	59	HCC	3	14	250	0	9.57*10^3^	610	22.41	TC+MMF	ADV	0.10	167.51	113.51
15	B	44	BLC	8	22	0.02	0	NA	1307	20.55	TC+MMF	ETV	9.13	13.77	36.17
16	O	36	BLC	5	19	250	0	1.96*10^3^	633	22.65	TC+MMF	ADV	30.70	26.94	33.25
17	A	62	HCC+BLC	20	19	0.01	0	2.18*10^7^	390	20.90	SR+CyA	ETV	21.62	16.91	21.62
18	A	60	BLC	10	11	250	0	<100	790	24.44	TC+MMF	ETV	32.59	30.5	56.49
19	B	38	HCC	3	8	250	0.09	<100	941	22.66	TC+MMF	ETV	0.00	11.95	0.35
20	O	42	BLC	13	NA	0.02	0.35	NA	1575	23.81	TC+MMF	LAM	13.98	10.46	10.57
21	O	37	B-FHF	13	25	250	0	NA	1532	19.84	TC+MMF	ETV	15.04	19.18	27.67
22	B	61	BLC	1	10	90.28	14.37	3.35*10^3^	945	26.89	SR	ETV	14.14	15.45	9.74
23	O	57	BLC	5	14	250	0.21	6.95*10^6^	394	24.80	TC+MMF	ETV	36.87	19.58	7.536
24	A	42	HCC	7	3	250	0.42	1.39*10^3^	972	19.96	TC+MMF	LAM	15.36	14.55	14.55
25	B	47	HCC+BLC	10	7	250	0.26	<100	397	26.30	TC+MMF	ETV	17.86	15.83	11.76
26	B	39	B-FHF	7	26	250	0.51	7.14*10^7^	501	26.17	TC+MMF	ETV	26.12	28.38	12.85
27	AB	56	HCC+BLC	33	14	114.16	1.42	NA	1853	24.22	TC	ADV	2.43	0.68	0.68

*, results of qualitative detection from primary hospital.

B-FHF, hepatitis B related fulminant hepatic failure; BLC, hepatitis B related liver cirrhosis; HCC, hepatocellular carcinoma; NAs, nucleos(t)ide analogues; LAM, lamivudine; ADV, adefovir dipivoxyl; ETV, entecavir; TC, tacrolimus; SR, sirolimus; MMF, mycophenolate mofetil; CyA, cyclosporine A; f/u, follow-up; NA, not applicable.

### The effects of vaccination

**Fig 2:**
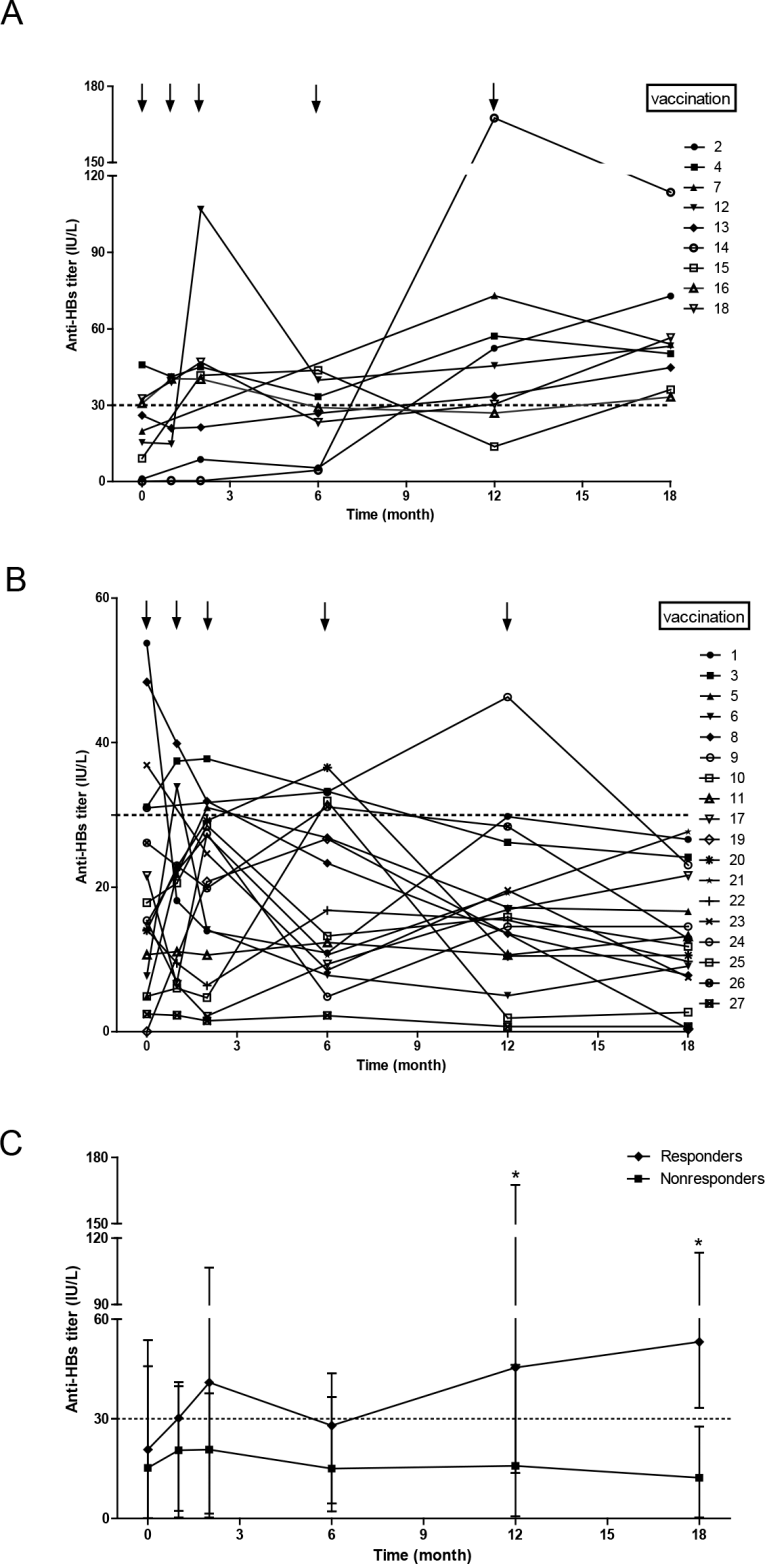
The dynamic changes of anti-HBs titers during the vaccination and follow-up periods. (A) Responders, (B) non-responders and (C) overall kinetics of both groups. *Mann–Whitney U-tests, P<0.01.

All patients completed the full vaccination course and the average anti-HBs titer before vaccination was 19.86±14.80 IU/L (median 15.38 IU/L; range 0–53.76 IU/L). Upon completion of the trial, nine patients were classified as responders with an average anti-HBs titer of 57.14±22.75 IU/L (median 53.16 IU/L; range 33.25–113.51 IU/L), giving a response rate of 33.3% (9/27). Nevertheless, anti-HBs titer in 18 non-responders gradually decreased to 12.66±8.24 IU/L (median 12.31 IU/L; range 0.35–27.67 IU/L) by the end of our study. The dynamic changes of anti-HBs titer in both responders and non-responders are shown in Fig 2. Compared with the non-responder group, the responder group showed its significant difference in anti-HBs titer after the fourth vaccination (P<0.01). In addition, anti-HBs titers of responders increased dramatically after the fifth vaccination compared to their initial titers at enrollment (P<0.05), but no significant change was found in non-responders’ titers during the course. Double-dose vaccination was well-tolerated except for the occurrence of a self-limited fever in one subject. During the study period, all participants were free of HBV recurrence and rejection.

### Predictors of response

To elucidate the characteristics for defining a responder, clinical data from responders and non-responders were investigated (Table 2). Patient factors such as age, etiology of liver diseases, virology, MELD score, HBV history, BMI, serum creatinine, immunosuppressant regiment, NAs and white blood cell count and classification were not significantly different between two groups. There was a trend that a longer interval between LT and vaccination existed in responders. The interval time of all responders was more than 600 days, while 11 of the non-responders exceeded that length of interval time (P = 0.059). Obviously, the rate between lymphocyte number and eosinophil number (LY/EO) of responders was significantly lower than those of non-responders at the start of vaccination (P = 0.019). The majority of non-responders (11/18, 63.64%) had high LY/EO rates (>15), while most responders (8/9, 88.89%) had low LY/EO rates (≤15). On the other hand, 91.7% (11/12) of patients with a high LY/EO rate were non-responders (Table 3).

**Table 2:** Comparison between responders and non-responders.

Factor	Responder(n = 9)	Non-responder(n = 18)	*P* Value
**At time of LT**			
Age (years)	46.0 (36–60)	47.5 (26–64)	1.000
Etiology (with cirrhosis)	8 (88.89%)	12 (66.67%)	0.336
Positive HBsAg	7 (77.78%)	16 (88.89%)	0.582
Positive HBV DNA	6 (66.67%)	7 (38.89%)	0.236
MELD score	19 (3–24)	24 (3–26)	0.803
HBV history (years)	8 (3–23)	11.5 (1–23)	0.860
Interval since LT (>600 days)	9 (100.00%)	11 (61.11%)	0.059
**At time of enrollment**			
Age (years)	48.0 (38–62)	51.5 (27–67)	0.781
BMI (kg/m^2^)	23.72±2.93	24.06±3.01	0.782
Anti-HBs (IU/L)	19.87(0.10–45.84)	15.20(0.00–53.76)	1.000
Serum creatinine (umol/L)	91±17	90±15	0.875
WBC(*10^9/L)	5.62±1.17	5.83±1.46	0.703
NEUT%	60.83±7.51	55.42±8.86	0.129
LY%	27.09±6.40	33.01±10.22	0.126
MO%	7.37±2.18	7.56±2.54	0.846
EO%	4.36±4.40	3.61±5.19	0.714
BASO%	0.34±0.12	0.42±0.19	0.316
LY/EO (≤15)	8 (88.89%)	7 (38.89%)	0.019
**During vaccination**			
Smoking	2 (22.22%)	3 (16.67%)	1.000
HBIG administration(>6 times)	2 (22.22%)	4 (22.22%)	1.000
Single immunosuppression	3 (33.33%)	5 (27.78%)	1.000
Concentration of TC(ug/L)	6.44±2.94	5.79±2.17	0.560^†^
NAs (LAM/ADV/ETV)	2/3/4	5/3/10	0.617

†Data were missing in five cases due to their single immunosuppression regimen.

MELD, model for end-stage liver disease; BMI, body mass index; WBC, white blood cell; NEUT, neutrophil; LY, lymphocyte; MO, monocyte; EO, eosinophil; BASO, basophil; TC, tacrolimus; LAM, lamivudine; ADV, adefovir dipivoxyl; ETV, entecavir.

**Table 3:** Distribution of LY/EO ratio in responders and non-responders.

	Low LY/EO ratio (≤15)	High LY/EO ratio (>15)	Total
Responders	8	1	9
Non-responders	7	11	18
Total	15	12	27

LY, lymphocyte; EO, eosinophil.

## Discussion

Though numerous studies regarding active immunization in transplanted patients have been reported with controversial results, few prospective trials were carried out in accordance with a specific vaccination program [8–10]. Here we gave each subject double-doses of vaccine (40 μg) by intramuscular injection at 0, 1, 2, 6 and 12 months after enrollment, with a response rate of 33.3% (9/27). In other words, approximately 33.3% of post-transplant patients could employ a cheaper method to maintain relatively safe antibody titers. The cost of HBIG (400U) would be roughly $100, whereas that of the vaccine double-doses (40 μg) was nearly $35 in China. Compared with other centers’ regimens, our vaccination protocol was varied with respect to subject selection, type and dose of vaccine, time of commencement and frequency of vaccination, vaccination route, combination with HBIG and/or NAs, and immunosuppressive status at the time of vaccination.

Decreased vaccine response is associated with patient factors such as smoking, obesity, diabetes, older age (>50 years), suffering from chronic hepatitis B [11]. However, we failed to find differences in these factors between responders and non-responders, partly due to our small cohort. Previous study indicates patients with chronic HBV infection react poorly to vaccines as they have developed a tolerance to HBV antigen [12]. Nevertheless, the majority of responders were chronic HBV carriers in this trial. As we know, the liver is the biggest immune organ and plays a critical role in immune responses. Inside the liver is located a huge amount of sinusoidal endothelial cells, stellate cells and liver parenchymal cells, acting as antigen-presenting cells. A healthy liver helps to keep the balance between naive and memory cellular populations and trigger formation of a memory-like T-cell differentiation [13]. With the improvement in nutrition and immunity, post-transplanted recipients gradually restore reactivity against specific third-party antigens, like the HBV vaccine, which may be one reason that accounts for response in chronic HBV carriers. Moreover, adoptive immune transfer of HBV-specific immune response from donors could be part of the explanation, and memory T lymphocytes and memory B lymphocytes play the most important role in this process [14]. Unfortunately, we lost the anti-HBs titers of most donors, thus we are unable to explore the association between donor factors and vaccination response. Furthermore, patients in the present trial maintained a certain titer of anti-HBs during the vaccination period. On one hand, the supplement of HBIG protected subjects from HBV reinfection during the study; on the other hand, formation of HBIG/HBsAg complexes might increase vaccine immunogenicity [15].

Several reports have identified that the HBV vaccine type is of critical importance for the final immune response outcome. The second-generation recombinant HBV vaccines constructed in yeasts transfected with HBV-DNA sequences coding for the small HBsAg were developed in the mid-1980s [16]. Although it has more than 90% efficiency in the general population, recombinant HBsAg was effective in only 7.7–29.6% of transplanted recipients. To enhance the effectiveness of the vaccine [17], some adjuvants were added, like 3-deacylated monophosphoryl lipid A and Quillaja saponaria [18]. However, their efficacy and safety still require clarification. The third generation vaccine, Sci-B-Vac complex vaccine, containing S, pre-S2 and/or pre-S1, has more immunogenic advantages by eliciting a faster and more intense antibody response as well as an increased response rate [19]. Since the adjuvant vaccine and the third generation vaccine were still not commercially available in most parts of China, we adopted double-doses of monovalent vaccine Engerix-B in this study. Meanwhile, adding doses of vaccine was proved to be more responsive and safe enough in post-transplant patients.

Even though a longer interval time between transplantation and vaccination enable recipients to minimize immunosuppression maintenance therapy thus leading to a higher vaccination response rate, HBV reinfection is rare beyond 18 months after grafting and the value of prevention decreases with the declining risk of HBV recurrence. Lu SC et al suggested it was reasonable to initiate reconstruction of active immunity 18 months after transplantation, while some experts believe two years would be better for the consideration of subjects’ safety [15, 20]. To date, no consensus has been reached on the commencement time of vaccination, though most trials enrolled patients at least one year after transplantation. In the aspect of inoculation frequency, Peter Starkel et al thought at least four injections of vaccine were needed to mount the immune response in immunocompromised individuals [21]. Similarly, we found responders and non-responders showed their significant difference in anti-HBs titers after the fourth vaccination (P<0.01).

It is conceivable that intradermal delivery of HBV vaccines is more immunogenic than intramuscular vaccination. Epidermis is rich with antigen-presenting cells, making it more likely to induce a strong T- and B-cell response [22]. Nevertheless, the intradermal route is inclined to cause more regional pain and discomfort and vaccine leakage may occur due to the irresistible dodge of patients during the inoculation. As a result, we administered the traditional intramuscular route in our trial, because it would increase the likelihood of subjects’ participation and continued clinical surveillance.

Furthermore, immune status of the subjects is crucial to their HBV vaccine response. Though we found no significant difference in immunosuppressive regimen between the above two groups, according to literature reports, the immunosuppressant’s serum concentration could not exactly reflect the immune status of patients [23]. Therefore, novel methods were developed aimed at precisely assessing patients’ immunity, in order to give a hand in minimizing the dose of immunosuppressive agents, reduce the risk of infection, and lessen financial burden. M. Usui et al reported that notably higher levels of TNF-α and INF-γ were found in the culture supernatant of responders’ T cells after adding HBV vaccine [19]. Hiroyuki Tahara et al used mixed lymphocyte reaction assays for evaluation of immune status, and found that post-transplant recipients with a well-maintained response to the third-party stimulus always achieved a sustained immune response to the vaccine [24]. To directly detect cellular immune function, the ImmuKnow assay was applied by measuring the production amount of ATP. However, further study is needed to confirm the value of ImmuKnow in predicting vaccination response.

According to our study, we found a negative correlation between lymphocyte-to-eosinophil (LY/EO) ratio and subjects’ immunoreaction to vaccination. The majority of non-responders (11/18, 63.64%) had high LY/EO rates (>15), while most responders (8/9, 88.89%) had low LY/EO rates (≤15) (P = 0.019). On the other hand, 91.7% (11/12) of patients with a high LY/EO rate were non-responders, which suggested there was little change to induce anti-HBs antibodies in patients with high LY/EO rates. In other words, more attention should have been paid to patients with low LY/EO rates when we were selecting vaccination candidates. Yenigun A et al discovered that the LY/EO ratio was significantly lower in children with allergic rhinitis than the normal control, and also is negatively correlated with nasal polyposis recurrent, indicating that this ratio reflected the immunity of subjects towards exogenous antigens [25–27]. The lower the ratio, the stronger the reaction might be. Zhang XY et al reported the LY/EO rate can accurately predict eosinophilic asthma in patients with persistent uncontrolled asthma despite treatment, which suggested this indicator was more stable and resistant to the immunoregulator’s effect [28]. Wong TW et al revealed eosinophils was capable of regulating humoral immunity via its impact on B cell homeostasis and proliferation upon activation in both mice and humans [29]. More and more research is proving the interactions between eosinophil and lymphocyte, demonstrating the role eosinophil plays in connecting innate immunity with adaptive immunity [30, 31]. Accordingly, LY/EO rate would be a potential predictor in selecting suitable transplanted recipients for accepting vaccination as a preventive strategy against HBV recurrence. However, the ratio was based on a small cohort with weak statistical power, and additional well-designed prospective studies are needed to determine its worth.

In conclusion, we conducted a prospective study on a procedural active immunization in patients transplanted for HBV related liver diseases, which was proved to be feasible, effective, dependable, and economical. We also found that subjects with low LY/EO rates are more likely to benefit from vaccination. In other words, LY/EO rates is a potential ‘easy-to-use’ biomarker in the early identification of best candidates for HBV vaccine administration. However, a well-designed multicenter study with greater enrollment of patients should be performed to further explore whether more immunogenic vaccines or alternative schedules of vaccination could improve these results.

## Supporting information

S1 File The TREND statement checklist. (PDF)

S2 File The protocol in Chinese. (DOC)

S3 File The protocol in English. (DOC)

S4 File The primary ethical approval in Chinese. (PDF)

S5 File The translation of primary ethical approval in English. (DOCX)

S6 File The data set of clinical trial result. (SAV)
