# Supplementary material for: Active immunization in patients transplanted for hepatitis B virus related liver diseases: A prospective study
Source: PLoS One. 2017 Nov 16;12(11):e0188190. doi: 10.1371/journal.pone.0188190 (PMC5690662; doi:10.1371/journal.pone.0188190)
Supplement: S3 File — (DOC) [file pone.0188190.s003.doc]

Title：Prospective study on hepatitis B vaccination in patients transplanted for HBV related liver diseases

Aims：To prospective study the safety and efficiency of hepatitis B vaccine in preventing hepatitis B recurrence in post-transplant patients.

Inclusion criteria:

1. The age of patients should be ranged from 18 to 79 years old, and there is no limitation in gender.

2. Patients transplanted for HBV related liver diseases or received hepatitis B core antibody (HBcAb) positive donor livers.

3. Patients who had received the transplant for more than 12 months.

4. No evidence of HBV recurrence, that is seronegative for HBsAg and HBV-DNA.

5. Normal liver function is required. (The parameters of laboratory examination should be less than double of the reference range).

6. There was no severe episode of rejection during the vaccination and no infection occurred in the last month.

7. Patients were in a mild state of immunosuppression, taking single immunosuppressant or multiple immunosuppressants in low doses and steroid was weaned off for at least 6 months.

8. Patients should sign informed consents after full understanding of the trial.

Exclusion criteria：

1. Post-transplant patients with tumor recurrence.

2. Occurrence of biliary complications: biliary stricture, bile leakage, biliary sludge formation, ischemic changes of biliary tract.

3. Occurrence of vascular complications: stenosis of hepatic artery, portal vein or inferior vena cava, hemorrhage, thrombosis.

4. Coinfection with hepatitis C virus, hepatitis D virus or human immunodeficiency virus.

5. Recipients with renal insufficiency which requires regular dialysis treatment.

6. Patients used to be diagnosed as organic cardiopathy or arrhythmia. 7. Women with positive result of pregnancy test or in breast feeding period, or the young couple going to give birth to a baby in the near future.

8. Patients used to be hypersensitive to any ingredient of our vaccine.

Exit criteria:

1. Patients pass away during the trial, do not get inoculation according to our schedule or require to exit the trial.

2. Occurrence of serious vaccine-related adverse events.

3. Episode of acute or chronic rejection.

4. Recurrence of hepatitis B, which was proved by positive hepatitis B surface antigen (HBsAg) or HBV-DNA in serum.

5. Pregnancy during the study period.

Subject number and enrollment time:

100 eligible post-transplant patients will be recruited from September 1^st^, 2013 to December 31^st^, 2014.

Type and dose of vaccine:

We are going to adopt double-dose (40ug) of recombinant hepatitis B surface antigen (HBsAg) vaccine (Engerix-B, GSK, 20ug/1.0ml) for vaccination by intramuscular injection.

Immunization program:

Participants are going to get vaccination at month 0, 1, 2, 6 and 12 after enrollment.

HBIG administration:

All subjects will be administrated HBIG 4000U by intravenous infusion during the anhepatic phase, and get HBIG 2000U by intravenous infusion daily during the first post-transplant week. The titer of anti-HBs antibodies will be maintained higher than 200 IU/L during the first three months after transplantation. And then the level of anti-HBs antibodies will be kept above 100 IU/L. The anti-HBs titer will be tested on the day prior to the inoculation during the vaccination program. If the serum anti-HBs titer was lower than 30 IU/L, the participant would receive intramuscular injections of HBIG (400 IU/injection) at least 2 weeks after the inoculation.

Oral nucleos(t)ide analogues (NAs) usage：

Patients should take oral NAs once he or she was diagnosed with HBV related liver diseases. Patients received graft from hepatitis B core antibody (HBcAb) positive donor should take oral NAs after transplantation. Oral NAs should be continued indefinitely during the study

Data collection:

Height, weight, living habit (smoking and alcohol), blood routine examination, coagulation routine, liver function, Basal metabolism and biochemical, immunosuppressant concentration, titer of HBsAg, anti-HBs antibody (HBsAb), HBeAg, anti-HBe antibody (HBeAb) and anti-HBc antibody (HBsAb), HBV-DNA copies, B ultrasound of liver.

Responder classification:

1. The increase in antibody titer is not out of the administration of HBIG during the vaccination.

2. Anti-HBs titer greater than 30 IU/L was maintained for up to 6 months without using HBIG and vaccine during the follow-up.

End-point of study:

Completed the procedural inoculation and be followed up for at least 6 months.
