# Supplementary material for: Active immunization in patients transplanted for hepatitis B virus related liver diseases: A prospective study
Source: PLoS One. 2017 Nov 16;12(11):e0188190. doi: 10.1371/journal.pone.0188190 (PMC5690662; doi:10.1371/journal.pone.0188190)
Supplement: S4 File — (PDF) [file pone.0188190.s004.pdf]

中山大学附属第一医院医学伦理委员会

批复件

伦审[2013]102 号　　　　　　　　审议日期：2013 年 8 月 20 日

审议项目	影响移植物存活相关感染性疾病的诊治策略			
负责科室	器官移植科	项目负责人	何晓顺	
出席委员	性别	单位	专业	签名
余学清	男	中山大学附属第一医院肾内科	医学	
饶从志	男	中山大学附属第一医院科研处	科研管理	
王长希	男	中山大学附属第一医院肾移植科	医学	
王卓青	男	中山大学附属第一医院科研科	科研管理	
苏乔	女	中山大学附属第一医院动物实验中心	兽医学	
杨威	男	中山大学附属第一医院药学部	药学	
张武军	男	中山大学附属第一医院医务科	医疗管理	
金宇	女	中山大学公共卫生学院妇幼卫生系	应用心理学	
徐艳文	女	中山大学附属第一医院生殖医学中心	医学	
章海山	男	中山大学伦理学教研室	伦理学	
蒋小云	女	中山大学附属第一医院儿科	医学	
曾志荣	男	中山大学附属第一医院消化内科	医学	
蔡世荣	男	中山大学附属第一医院胃肠胰外科	医学	
蔡海宁	男	广东经纶律师事务所	律师	

已提交审阅的材料	1. 医学伦理委员会项目评审受理表<br>2. 医学研究项目伦理审查申请书<br>3. 知情同意书<br>4. 研究方案<br>5. 国家高技术研究发展计划（863 计划课题任务书）
投票结果	1.同意（10）人；2.作必要的修改后同意（0）人；<br>3.修改后再报会议审议（0）人；4.不同意（0）人；<br>5.终止或暂停先前已批准的试验（0）人。

医学伦理委员会意见：

1、经会议审议，器官移植科何晓顺提交的"影响移植物存活相关感染性疾病的诊治策略"，符合医学伦理原则及道德要求，同意研究者按本研究方案开展研究。

2、在研究过程中，应遵守国际《赫尔辛基宣言》及我国卫生部门制定的《涉及人的生物医学研究伦理审查办法（试行）》与 GCP 等相关的伦理原则、道德标准及相关的法律、法规、常规、制度等。如对研究方案或知情同意书进行修改应及时向医学伦理委员会书面报告，所涉及的伦理问题均应提交医学伦理委员会再审议。

主任委员（签字）：

批复日期：2013 年 8 月 20 日

声明：本委员会仅对审查与备案的临床研究项目中涉及的伦理与道德问题负责。

联系地址：广州市中山二路 58 号，中山大学附属第一医院医学伦理委员会，邮编:510080

联系电话：020-87334871、020-87332200 转 8035　　传　真：020-87333122
