# Supplementary material for: Active immunization in patients transplanted for hepatitis B virus related liver diseases: A prospective study
Source: PLoS One. 2017 Nov 16;12(11):e0188190. doi: 10.1371/journal.pone.0188190 (PMC5690662; doi:10.1371/journal.pone.0188190)
Supplement: S5 File — (DOCX) [file pone.0188190.s005.docx]

Clinical Research Ethical Approval by the First Affiliated Hospital of Sun Yat-sen University

Medical Ethics Committee (Translation edition)

Project No.: 2013【102】 Date: August 20^th^, 2013

Name of research protocol：Strategy for diagnosis and therapy of infective diseases influencing graft survival.

Department: Organ transplant Principle Investigators: Xiaoshun He

Conference Attendance

| Name | Gender | Work Unit | Major | Signature |
| --- | --- | --- | --- | --- |
| Xueqing Yu | Male | Department of Nephrology | Medicine |  |
| Congzhi Rao | Male | Scientific Research Department | Scientific Research Management |  |
| Changxi Wang | Male | Renal Transplant Department | Medicine |  |
| Zhuoqing Wang | Male | Scientific Research Department | Scientific Research Management |  |
| Qiao Su | Female | Experimental Animal Center | Veterinary Medicine |  |
| Wei Yang | Male | Department of Pharmacy | Pharmacy |  |
| Wujun Zhang | Male | Medical Office | Medical Management |  |
| Yu Jin | Female | School of Public Health | Applied Psychology |  |
| Yanwen Xu | Female | Center for Reproductive Medicine | Medicine |  |
| Haishan Zhang | Male | Department of Ethics | Ethics |  |
| Xiaoyun Jiang | Female | Pediatrics | Medicine |  |
| Zhirong Zeng | Male | Gastroenterology | Medicine |  |
| Shirong Cai | Male | Gastrointestinal Surgery | Medicine |  |
| Haining Cai | Male | Jinglun Law Office | Law |  |

Material Submitted for Review:

1. Medical ethics committee project review acceptance form

2. Medical research project ethical review application report

3. Informed consent

4. Clinical trial protocol

5. National High Technology Research and Development Program (863 program project mission statement)

Result of Voting:

Agree: 10 votes

Agree after revised: 0 votes

Retrial after revised: 0 votes

Disagree: 0 votes

End or pause the approved clinical trials: 0 votes

Opinion of the Medical Ethics Committee:

1. After review meeting, we believe the project “Strategy for diagnosis and therapy of infective diseases influencing graft survival”, submitted by Xiaoshun He from Organ Transplant Department, is in line with the principles of medical ethics and moral requirements, and come to an agreement that the researcher could conduct the clinical trial according to the research protocol.

2. The trial should be conducted in accordance with the Declaration of Helsinki principles,

Human Involving Biomedical Research Ethics Review Approach enacted by Chinese Government, GCP related ethical principles, code of ethics, related laws and regulations. If any modification was made in the part of informed consent or clinical trial protocol, a report should be sent to the medical ethics committee, and the ethical issues involved should be re-reviewed by the medical ethics committee.

Chairman: Xueqing Yu

Date: August 20^th^, 2013

Statement: Our committee will be responsible for the ethical and moral issues in the clinical research project that was reviewed and recorded.

Medical Ethics Committee, The First Affiliated Hospital of Sun Yat-sen University

Address: No.58 Zhongshan 2^nd^ Road, Guangzhou 510080, China

Tel: 020-87334871 or 020-87332200 then dial 8035

Fax: 020-87333122
