# Supplementary material for: Active immunization in patients transplanted for hepatitis B virus related liver diseases: A prospective study
Source: PLoS One. 2017 Nov 16;12(11):e0188190. doi: 10.1371/journal.pone.0188190 (PMC5690662; doi:10.1371/journal.pone.0188190)
Supplement: Supplementary file 5 [file pone.0188190.s002.doc]

中山大学附属第一医院医学伦理材料

项目编号：伦审[2013]102 号

会议时间：2013 年 08 月 20 日

项目名称：影响移植物存活相关感染性疾病的诊治策略：肝移植术后主动免疫的前瞻性研究

□多中心，中心单位　□多中心，分中心　√□单中心

项目类型：□药物临床IV期试验　□动物实验

√□临床观察研究　□其它

项目负责人：何晓顺

所在科室：器官移植科

中山大学附属第一医院伦理委员会制

目 录

1. 医学伦理委员会项目评审受理表……………………………..….1

2. 医学研究项目伦理审查申请书/实验动物伦理审查申请书…….2

3. 知情同意书……………………………………………………11

4. 研究方案………………………………………………………14

5. 病例报告表……………………………………………………16

医学伦理委员会项目评审受理表

受理号：102

<table>
<tr><td>项目名称</td><td colspan="5">影响移植物存活相关感染性疾病的诊治策略：肝移植术后主动免疫的前瞻性研究</td></tr>
<tr><td>项目负责人</td><td>何晓顺</td><td>所在科室</td><td>器官移植科</td><td>联系电话</td><td>87306082</td></tr>
<tr><td>研究类别</td><td colspan="5">□药物临床试验 □医疗器械临床验证 □人类辅助生殖技术
□医疗新技术 □动物实验 √□临床观察研究 □其它</td></tr>
<tr><td>经费来源</td><td colspan="5">国家高技术研究发展计划（863 计划）专项经费（2012AA021008）</td></tr>
<tr><td>研究目的与意义</td><td colspan="5">感染、排斥反应和心脑血管疾病是当前影响移植受体预后的主要原因。在器官移植受体中，由于免疫抑制剂的应用，各种类型感染的发生率明显增高，而且临床表现不典型，与非移植受体感染谱、感染特点、诊断和治疗上均存在较大的差异，这些因素均极大地制约我国器官移植受体感染并发症的治疗效果。为此，我们需通过该研究，制定符合我国国情的器官移植受体感染并发症的临床诊疗规范，明确器官移植受体感染并发症的特点，发现感染并发症的易感人群，早期诊断感染并发症，制定器官移植术后乙肝复发的防治新策略，显著提高器官移植受体感染并发症的治愈率，改善移植受体的短期和长期预后。</td></tr>
</table>

**申报资料清单**	□国家食品药品监督管理局（SFDA）或其它上级主管部门批件<br>□药品注册证 √□临床试验方案 √□受试者知情同意书 □研究者手册<br>√□病历报告表 □质量检验报告 □研究者名单及简历 □研究合作协议书<br>□中心伦理委员会审批件 □试验产品已有的有效性和安全性资料 □产品使用说明书 □招募广告 □企业法人营业执照<br>□GMP 证 □产品生产许可证 □其他
**科室意见**	
**项目主管部门意见**<br>（此栏由药物临床研究基地或科研等主管部门填写）	
**伦理委员会意见**	□ 资料齐全，同意受理。<br>□ 资料不齐全，补充后受理。<br>□ 其他：

中山大学附属第一医院

医 学 研 究 项 目 伦 理 审 查

申 请 书

项目名称：影响移植物存活相关感染性疾病的诊治策略：肝移植术后主动免疫的前瞻性研究

起止日期：2013 年 9 月至 2015 年 12 月

申请科室：器官移植科

项目负责人：何晓顺

通讯地址：广东省广州市中山二路 58 号

邮政编码：510080

联系电话：020-87306082

传　真：020-87306082

电子信箱：gdtrc@163.com

申请日期：2013 年 8 月 1 日

中山大学附属第一医院医学伦理委员会制

研究目的与意义：
20 世纪末外科技术和器官保存技术的进步，以及新型免疫抑制剂的应用已使器官移植受体的预后得到了显著的提高。外科并发症已不再是影响移植受体存活率的主要因素，而感染、排斥反应和心脑血管疾病成为了当前影响移植受体预

后的主要原因。在器官移植受体中，由于免疫抑制剂的应用，各种类型感染的发生率明显增高，但在感染谱、临床表现、诊断和治疗上与非移植受体均存在较大的差异，这些差异给临床诊断和治疗带来极大的困难。而关于我国器官移植受体术后感染的易感因素，目前尚没有大宗临床报道以指导临床医生确定感染并发症的易感人群。此外，移植器官的感染常常难以区别于排斥反应等非感染性发热病因，传统的诊断方法亦无法做出快速、明确的诊断。另一方面，我国是乙肝大国，却少见我国关于器官移植术后乙肝复发预防和治疗的前瞻性临床研究。更重要的是，我国虽然器官移植数量已居全球前 3 位，目前仍没有出台一部器官移植感染并发症的诊疗规范，无法在全国范围内规范感染并发症的诊断与治疗。所有这些因素均极大地制约我国器官移植受体感染并发症的治疗效果，影响移植受体的存活率和生存质量。

为此，本课题将围绕如何克服上述制约因素开展以下四大方面的研究：（1）器官移植受体与非移植受体感染并发症的对比研究；（2）器官移植受体术后感染的风险评估研究；（3）器官移植术后感染并发症的新型诊断方法研究；（4）乙肝复发的预防与治疗新策略研究。在这些研究的基础上，组织相关领域的专家制定我国器官移植受体感染并发症诊疗规范。

上述研究的开展，有望实现以下目标：制定符合我国国情的器官移植受体感染并发症的临床诊疗规范，明确器官移植受体感染并发症的特点，发现感染并发症的易感人群，早期诊断感染并发症，制定器官移植术后乙肝复发的防治新策略，显著提高器官移植受体感染并发症的治愈率，改善移植受体的短期和长期预后。
**研究所需费用及来源：**<br><br>300 万元，来自国家高技术研究发展计划（863 计划）专项经费。

主要研究者及参与研究者的简介：

序号	姓名	性别	出生日期	证件类型	证件号码	技术职称	职务	专业	投入本课题的全时工作时间（人月）	人员分类	在课题中分担的任务	是否有工资性收入	工作单位
1	何晓顺	男	1964-08-30	身份证	440102196408303230	正高级	副院长	临床医学与医学技术类	32	课题负责人	课题设计、组织实施	是	中山大学附属第一医院
2	郭志勇	男	1981-11-01	身份证	440181198111011514	初级	无	临床医学与医学技术类	40	课题骨干	研究设计	是	中山大学附属第一医院
3	朱晓峰	男	1960-11-20	身份证	440102601120327	正高级	副主任	临床医学与医学技术类	32	课题骨干	细胞免疫功能检测	是	中山大学附属第一医院
4	王东平	男	1965-10-05	身份证	410103196510053777	副高级	副主任	临床医学与医学技术类	32	课题骨干	芯片制作	是	中山大学附属第一医院
5	鞠卫强	男	1975-08-24	身份证	352101197508246534	中级	无	临床医学与医学技术类	32	课题骨干	数据收集	是	中山大学附属第一医院
6	巫林伟	男	1978-06-05	身份证	352622780605001	中级	无	临床医学与医学技术类	32	课题骨干	统计分析	是	中山大学附属第一医院
7	邰强	男	1976-08-11	身份证	440102197608113233	中级	无	临床医学与医学技术类	32	课题骨干	统计分析	是	中山大学附属第一医院
8	胡安斌	男	1975-04-06	身份证	370321197504063915	副高级	无	临床医学与医学技术类	32	课题骨干	microRNA 检测	是	中山大学附属第一医院
9	马毅	男	1968-12-01	身份证	340104196812012055	正高级	无	临床医学与医学技术类	32	课题骨干	lncRNA 检测	是	中山大学附属第一医院
10	王国栋	男	1970-07-15	身份证	410722700715083	副高级	无	临床医学与医学技术类	32	课题骨干	易感基因检测	是	中山大学附属第一医院
11	孙丽莹	女	1967-05-28	身份证	510102196705286121	正高级	主任	临床医学与医学技术类	36	课题骨干	课题设计	是	天津市第一中心医院
12	曲伟	男	1981-11-13	身份证	21022519811113003X	初级	无	临床医学与医学技术类	32	课题骨干	实验分析	是	天津市第一中心医院
13	涂金鹏	男	1980-06-09	身份证	130102198006091518	初级	无	临床医学与医学技术类	32	课题骨干	样本处理	是	天津市第一中心医院
14	孙晓叶	女	1975-10-28	身份证	370631197510287527	初级	无	临床医学与医学技术类	32	课题骨干	数据分析	是	天津市第一中心医院
15	吴成林	男	1986-03-11	身份证	421181198603119115	其他	无	临床医学与医学技术类	40	其他研究人员	细胞培养	否	中山大学附属第一医院
16	邓斐文	男	1977-08-07	身份证	460025197708073315	其他	无	临床医学与医学技术类	40	其他研究人员	细胞培养	否	中山大学附属第一医院
17	李强	男	1984-02-27	身份证	452503198402272059	其他	无	临床医学与医学技术类	40	其他研究人员	免疫检测	否	中山大学附属第一医院
18	林建伟	男	1984-08-21	身份证	4452241984 0821397x	其他	无	临床医学与医学技术类	40	其他研究人员	标本收集、处理	否	中山大学附属第一医院
19	赵强	男	1986-12-27	身份证	130503198612270039	其他	无	临床医学与医学技术类	40	其他研究人员	CTL 构建	否	中山大学附属第一医院
20	张传钊	男	1987-12-15	身份证	445381198712157813	其他	无	临床医学与医学技术类	40	其他研究人员	病例随访	否	中山大学附属第一医院
21	饶伟	男	1982-09-13	身份证	12010419820913681X	初级	无	临床医学与医学技术类	32	其他研究人员	样本采集	是	天津市第一中心医院
22	曾志贵	男	1980-01-21	身份证	51112119800121281X	初级	无	临床医学与医学技术类	32	其他研究人员	数据分析	是	天津市第一中心医院

研究方法（包括试验设计及进行步骤、试验期限及进度、试验评估及统计方法等摘要）：

（一） 器官移植受体与非移植受体感染并发症的对比研究

组织国内多家器官移植中心研究对比肝移植受体与肝脏外科术后患者、SICU 非移植患者和消化内科终末期肝病患者感染并发症部位、感染谱、诊断方法、治疗方法、治疗效果与感染患者预后的特点，研究对比肾移植受体与肾脏外科术后患者、肾内科肾衰竭患者感染并发症部位、感染谱、诊断方法、治疗方法、治疗效果与感染患者预后的特点，总结器官移植受体感染并发症特点，为制定我国器官移植受体感染并发症诊疗规范奠定扎实的基础。

（二） 器官移植受体术后感染的风险评估研究

组织国内多家器官移植中心回顾分析患有感染并发症的移植受体的临床、供体、手术、免疫抑制方案、感染预防方案、生化指标等临床资料，明确我国器官移植受体感染并发症发生的危险因素；监测 T 细胞免疫功能，制定我国移植受体 Immuknow 正常值范围以及预测感染并发症的下限；通过基因多态性分析，发现我国移植受体发生常见致命性感染类型，如巨细胞病毒（CMV）感染等的易感基因。

（三） 器官移植术后感染并发症的新型诊断方法研究

制备器官移植术后感染患者不同标本的基因组 DNA 样品，利用 16S-rDNA 技术和比较基因组技术，研制我国器官移植受体常见感染细菌的 16S-rDNA 基因诊断芯片；研究感染和非感染器官移植受体外周血浆 microRNA（miRNA）和外周血单核细胞长链非编码 RNA（lncRNA）表达谱的差异，发现可以用于诊断感染并发症的新标志物。

（四） 乙肝复发的预防与治疗新策略研究

比较乙肝免疫球蛋白和单种核苷酸类似物联用方案，与两种核苷酸类似物联用方案在预防移植术后乙肝复发疗效的差异，前瞻性研究乙肝疫苗在预防器官移植术后乙肝复发的安全性和有效性；建立乙肝病毒（HBV）特异性细胞毒性 T 淋巴细胞（CTL）系，评估 HBV 特异性 CTL 治疗乙肝复发的安全性和有效性。

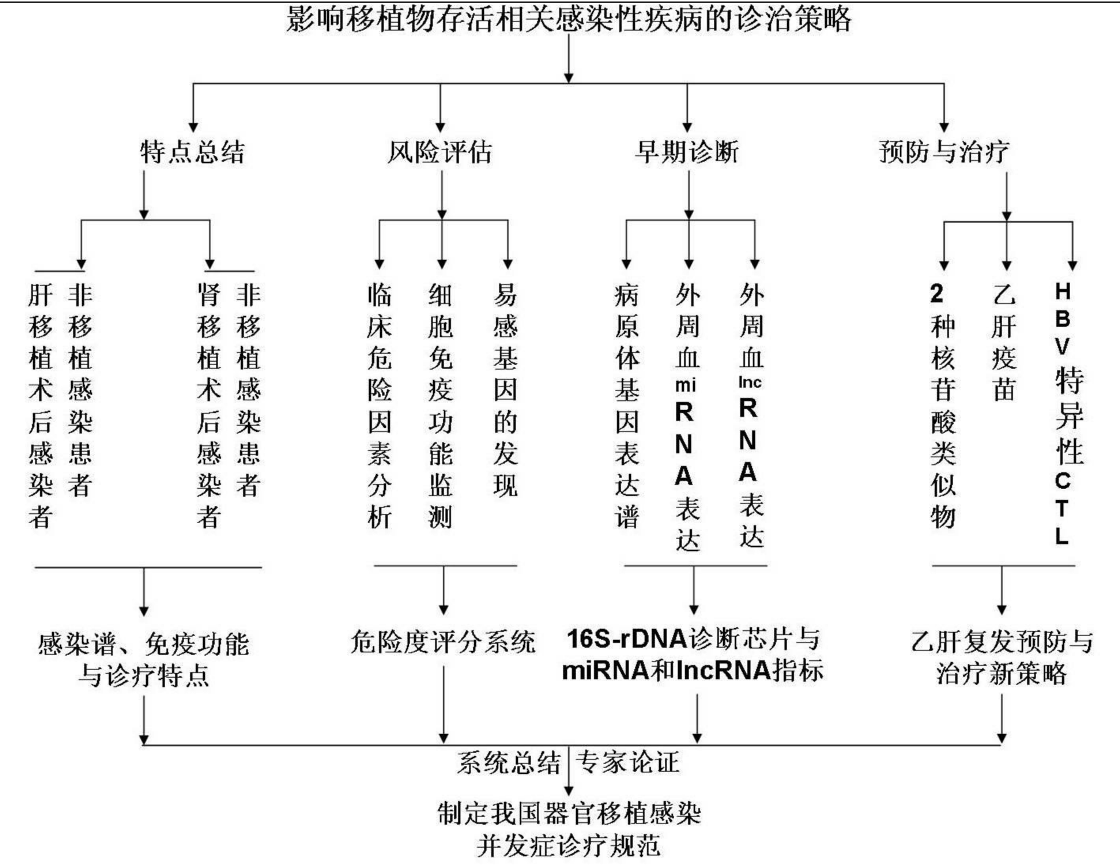

	工作计划	考核指标
第一年度	（1）开始收集感染组和非感染组器官移植受体的引流液、痰液、尿液和血清样本（2）筛查感染组和非感染组患者外周血的 miRNA 和 lncRNA 芯片表达差异；（3）收集、整理移植组和非移植组患者的感染谱和临床指标。	（1)建立感染受体的各类感染样本库；<br>（2）初步筛查感染受体的 miRNA 和 lncRNA 表达谱的差异；<br>（3)建立感染相关临床危险因素数据库。
第二年度	（1)开展常见感染细菌的 16s-rDNA 芯片的构建；（2）RT-PCR 的证实感染组和非感染组患者外周血的候选 miRNA 和 lncRNA；（3）统计分析确定器官移植术后感染的临床危险因素，撰写研究论文；（4）开始为 1 个前瞻性临床研究纳入病人；（5）开展感染患者的细胞免疫功能检测。	（1)证实外周血与移植后感染相关的 miRNA 和 lncRNA；<br>（2)撰写第 1-2 篇 SCI 论文，总结关于移植术后临床危险因素分析的结果；<br>（3)开始两种核苷酸类似物预防移植后乙肝复发的前瞻性研究的病人纳入。

第三年度	（1）在新发可疑感染患者中验证16s-rDNA 芯片的检测效能，撰写研究论文；（2）在新发可疑感染患者中验证候选 miRNA 和 lncRNA 的诊断和预测效能，撰写研究论文；（3）在大样本术后感染器官移植受体中发现易感基因，建立风险评分系统，并在新发可疑感染患者中验证其预测效能，撰写研究论文；	撰写第 3-6 篇 SCI 论文。
第四年度	（1）完成前瞻性临床研究的病人纳入、随访工作，撰写研究论文；（2）将研发的 16s-rDNA 芯片申请国家应用专利；（3）组织国内外专家制定我国器官移植受体感染并发症诊疗规范；（4）召开继续教育培训班，向国内其他单位开展推广相关应用技术；（5）课题总结、验收。	（1）撰写第 7-8 篇 SCI 论文，总结关于两种核苷酸类似物预防肝移植术后乙肝复发的前瞻性研究结果。<br>（2）为研发的 16s-rDNA 芯片申请国家应用专利 1 项。<br>（3）召开继续教育培训班 1 次。

实验中计量性资料用 X±SD 表示，数据应用 SPSS13.0 软件分析，均数之间比较应用 T 检验，医院感染率间比较应用列联表分析卡方检验，危险因素分析应用单因素或多因素方差分析。

受试者的总例数、入选标准和排除标准：

在 100 例乙肝相关性肝病肝移植术后患者中前瞻性研究乙肝疫苗在预防器官移植术后乙肝复发的安全性和有效性。

入组标准：

1、年龄为 18 至 70 岁,男女不限；

2、原发病为乙肝相关性肝病或接受单纯核心抗体阳性供肝的受体；

3、肝移植术后至少 12 个月；

4、无乙肝复发迹象，即血清 HBsAg（-）且 HBV DNA（-）；

5、肝功能正常或基本正常（指标升高程度不超过正常值 2 倍）；

6、接种时无排斥反应发生，无近期（1 月内）感染；

7、处于轻度免疫抑制状态，即服用单种免疫抑制剂，或小剂量联用两种免疫抑制剂，并停用激素至少半年。

8、充分理解实验研究过程并签署知情同意书。

排除标准：

1、患者出现肿瘤复发；

2、出现胆道并发症：胆道狭窄、胆漏、胆泥形成、缺血性改变；

3、出现血管并发症：肝动脉、门静脉或下腔静脉出现狭窄、出血、血栓形成；

4、合并 HCV、HDV、HIV 感染；

5、合并肾功能不全，需要透析治疗；

6、明确器质性心脏病或心律失常者；

7、妊娠试验阳性或哺乳期妇女，计划近期内生育的男女；

8、既往明确对疫苗任何成分存在超敏反应者。

退出标准：

1、患者死亡、不按程序接种疫苗或要求退出研究；

2、出现与疫苗接种有关的严重不良反应；

3、出现急性或慢性排斥反应；

4、出现乙肝复发，即血清 HBsAg（+）或 HBV DNA（+）；

5、研究期间意外怀孕。

研究中可能出现的不良反应或可能发生的不良事件及其处理对策：

研究中可能出现：

1、注射部位短暂的疼痛、发红、肿胀；

2、偶有疫苗接种点感染、皮下血肿；

3、乙型肝炎复发；

4、罕见的疫苗不良反应: 发热、头昏、恶心、肌痛、皮疹、肝功能异常。

处理对策为：<br>1、接种后留观 1 至 2 小时，必要时给予局部理疗；<br>2、出现感染时给予局部消毒，必要时给予抗生素治疗，出现血肿时，12 小时内给予冰敷，其后给予局部热敷；<br>3、退出本研究，抽血行乙肝病毒 DNA 变异型检测，并调整抗乙肝病毒方案；<br>4、监测患者病情变化，并给予对症支持处理，严重时退出该研究。
**同意遵循相关指南规定的伦理性原则的声明（包括接受伦理委员会提出的要求、提交研究进度报告、进行方案、知情同意书修改时应通知等）**<br>**本人及全体课题组成员同意遵循相关指南规定的伦理性原则，接受伦理委员会提出的要求，提交研究进度报告，进行方案及知情同意书修改时通知伦理委员会等。**<br>**项目负责人签名：**<br>**年 月 日**
**执行科室意见：**<br>**负责人签名：**<br>**年 月 日**
**医院行政主管部门意见：**

**负责人签名：**<br><br>**年　　月　　日**
**医院伦理委员会意见：**<br><br><br>**负责人签名（章）**<br><br>**年　　月　　日**

影响移植物存活相关感染性疾病的诊治策略
肝移植术后主动免疫的前瞻性研究
知情同意书

前言

肝移植术后乙肝复发影响着移植患者的长期生存率和生活质量。长期使用 HBIG 抗病毒治疗的费用高昂，而服用核苷类似物抗乙肝病毒有一定的副作用，并有因病毒突变而失效的风险。因此，重建肝移植术后患者对乙肝病毒的主动免疫力有着重大的意义。

研究目的

前瞻性研究接种乙肝疫苗在预防肝移植术后乙肝复发的安全性和有效性。

研究的内容和步骤

1．医生将询问您的病史及目前服用免疫抑制剂的详细情况，并对您进行全面的体格检查和一系列的有关检查，以明确您目前的身体状况。如您有疑问，医生将详细解释这些化验检查。在此全过程共需要取血约 30-50 毫升进行化验检查。对于育龄期妇女，将进行妊娠试验。

2．检查结束后，如果您适合参加此项研究，医生将按照预定的程序为您接种乙肝疫苗，接种程序为：0、1、2、6、12 月各接种一次疫苗。0 月为您参加该研究并接种第一针疫苗的时间。

3．在研究过程中，医生将于您每次接种前抽血检测乙肝 HBsAb 滴度，若您的 HBsAb 滴度低于 30 IU/L，医生将给你使用 HBIG 以预防乙肝复发。同时，医生将对您进行抽血化验检查，以明确您的身体状况，并对您进行相关健康指导。每次抽血约 20-30ml。

4．在整个实验过程中，您将持续服用恩替卡韦或其他核苷类似物抗乙肝病毒治疗，

如有不适，请及时联系医生。

5．对于女性患者，需要在研究期间采用避孕措施。对于在研究期间怀孕或准备怀孕的患者，您将不能继续参加本项研究。若您出现肿瘤复发、乙肝复发、免疫排除反应及对疫苗成分出现超敏反应，您将退出该研究。

6．在研究期间，如需要服用其它药物（包括中药），请您告诉医生，医生将给予建议。

7．在整个研究过程中，医生将根据您的实际治疗情况，进行定期的必要检查，以正确评估您的健康状况及疫苗接种效果。

参加本项研究的益处

1．在研究开始时，您将接受全面的体检和相关辅助检查，并对您的病情作出全面、明确的诊断。

2．在研究期间，医生将对您进行病情监测，以便及时发现并发症并采取治疗措施。

3．若成功接种，您将获得对乙肝病毒的主动免疫力，免去了长期使用 HBIG 带来的不便和经济负担，您的生活质量将进一步提高。

研究期间可能出现的不适和风险

1. 注射部位短暂的疼痛、发红、肿胀;

2. 偶有疫苗接种点感染、皮下血肿;

3. 乙型肝炎复发;

4. 罕见的疫苗不良反应: 发热、头昏、恶心、肌痛、皮疹、肝功能异常。

针对不良事件采取的措施

1. 接种后留观 1 至 2 小时，必要时给予局部理疗;

2. 出现感染时给予局部消毒，必要时给予抗生素治疗，出现血肿时，12 小时内给予冰敷，其后给予局部热敷;

3. 退出本研究，抽血行乙肝病毒 DNA 变异型检测，并调整抗乙肝病毒方案;

4. 监测患者病情变化，并给予对症支持处理，严重时退出该研究。

研究资料的保密性

所有的研究资料将是保密的，由医院和科室保存。您的姓名不会出现在任何此项研究的发表资料或报告中，您的病例记录将在研究人员在场的情况下，提供给相关人员用于证实从本项研究中收集的资料，而不涉及您的个人详细资料。

自愿参加

您参加本项研究是完全自愿的，如您不愿参加，不会在任何方面影响医生对您的治疗。您可以在任何时间以任何理由退出本项研究，而且仍然不会影响医生对您的治疗。

如果您决定不再参加本项研究，请与您的医生联系。医生将会解释退出本项研究的最好的方法。

如果您退出研究，将在退出之日进行与上次随访相同的血液化验和检查。

健康受损的赔偿

如果您由于参加这项研究健康受到损害，医院将对您进行治疗，并遵照中国的有关法规和有关临床观察病人补偿原则给予补偿，赔偿主体为疫苗供应单位，赔偿范围是根据药物与不良反应关系判定标准判定的由使用该疫苗导致（很可能有关或肯定有关）的不良反应引起的诊断和治疗费用，目的是为了给在研究中因疫苗因素而使健康受损的病人以适当的补偿。

联系

在本研究的过程中，如果您有关于研究情况或您权利的问题，或者您认为出现了与本研究有关的任何情况，请与_____医生联系，电话________。

受试者的同意

我是在充分了解以上内容后自愿参加这项研究的，我愿意与研究人员合作，认真完成本研究。

我可以在任何时候因任何原因退出此研究。

医生已给我这份知情同意书，并已详细解释了此研究的性质和目的，医生已给我时间询问有关此疫苗研究的问题，并已说明我可以随时退出研究。

我______（全名）自愿同意参加此临床研究。

受试者签名_____，日期：_年_月_日　联系电话：______

医生签名______，日期：_年_月_日　联系电话：______

（同意书一式签署两份，医生和受试者各保存一份）

乙肝相关性肝病肝移植术后主动免疫的前瞻性研究

目的：

前瞻性研究接种乙肝疫苗在预防肝移植术后乙肝复发的安全性和有效性。

入组标准：

（1）年龄为 18 至 70 岁，男女不限；

（2）原发病为乙肝相关性肝病或接受单纯核心抗体阳性供肝的受体；

（3）肝移植术后至少 12 个月；

（4）无乙肝复发迹象，即血清 HBsAg（-）且 HBV DNA（-）；

（5）肝功能正常或基本正常（指标升高程度不超过正常值 2 倍）；

（6）接种时无排斥反应发生，无近期（1 月内）感染；

（7）处于轻度免疫抑制状态，即服用单种免疫抑制剂，或小剂量联用两种或多种免疫抑制剂，并停用激素至少半年。

（8）充分理解实验研究过程并签署知情同意书。

排除标准：

（1）患者出现肿瘤复发；

（2）出现胆道并发症：胆道狭窄、胆漏、胆泥形成、缺血性改变；

（3）出现血管并发症：肝动脉、门静脉或下腔静脉出现狭窄、出血、血栓形成；

（4）合并 HCV、HDV、HIV 感染；

（5）合并肾功能不全，需要透析治疗；

（6）明确器质性心脏病或心律失常者；

（7）妊娠试验阳性或哺乳期妇女，计划近期内生育的男女；

（8）既往明确对疫苗任何成分存在超敏反应者。

退出标准：

（1）患者死亡、不按程序接种疫苗或要求退出研究；

（2）出现与疫苗接种有关的严重不良反应；

（3）出现急性或慢性排斥反应；

（4）出现乙肝复发，即血清 HBsAg（+）或 HBV DNA（+）；

（5）研究期间意外怀孕。

入组人数及入组时间：

100 例符合条件的移植术后患者；自 2013 年 9 月 1 日至 2014 年 12 月 31 日。

疫苗类型及剂量：

安在时（重组酵母乙型肝炎疫苗，GSK，20ug/1.0ml），每次肌注 40ug。

接种程序：

入组后 0、1、2、6、12 月，每月接种一次。

HBIG 使用：

术中无肝期使用 HBIG 4000U 静脉滴注，术后 1 周每天 HBIG 2000U 静脉滴注，术后 3 月维持 HBsAb 滴度大于 200IU/l，随后维持 HBsAb 滴度大于 100IU/l 直到接种前。接种过程中，每月复查 HBsAb 滴度 1 次（离 HBIG 使用或者疫苗接种至少 2 周），维持抗体滴度大于 30 IU/l。

抗病毒药物使用：

自确诊乙肝相关性肝病之日起，每日口服恩替卡韦或其他核苷类似物抗乙肝病毒治疗，直至主动免疫成功建立。接受单纯核心抗体阳性供肝的受体，自术后第 1 天起，每日口服恩替卡韦或其他核苷类似物抗乙肝病毒治疗，直至主动免疫成功建立。

随访过程检测指标：

身高、体重、生活习惯、血常规、出凝血常规、肝酶学、肝代谢、基础代谢生化、药物浓度、乙肝两对半定量、乙型肝炎病毒 DNA 定量、肝胆胰脾 B 超。

接种成功标准:

(1)疫苗接种过程中，抗体滴度升高，不能用 HBIG 的使用来解析；

(2)停用 HBIG 后，至少随访 6 月，抗体滴度持续高于 30IU/L。

研究终点：

按预定程序接种乙肝疫苗后至少随访半年。

课题号	编号	患者姓名缩写	
	□□□	□□□□	

影响移植物存活相关感染性疾病的诊治策略

乙肝相关性肝病肝移植术后主动免疫的

前瞻性研究

病例报告表

(Case Report Form)

研究中心名称：中山大学附属第一医院

受试者姓名：___________

注： 报告表为保密材料，仅限于组织和进行该临床试验的有关人员使用。内容的所有权和解释权归属于广州中山大学附属第一医院器官移植中心。未经上述单位同意任何人不得以任何（包括复印、影印、电邮、口头等）形式外传，如有违反者，将视程度轻重予以相应的责任追究，或诉诸于法律。

在正式填表前，请认真阅读下列填表说明

病例报告表填写说明

1. 请用黑色圆珠笔用力填写，如果填写出现错误，请用一道线划去错误，重新填入正确数据以及更正人姓名缩写和更正日期。不要掩盖填入的原始数据不要用橡皮擦、用改正液遮盖或划许多道线。
2. CRF 的每一页都须完成，所有项目均应填写。在“□”处填入“√”表示选择此项。如果此项“未做”则填入“ND”；“不能提供”或“不适用”则填入“NA”。
3. 所有表格上的日期都以“年/月/日”的形式表示， 包括患者的出生日期。如果不知道具体日期，请用“UK”表示，以“年/月/ UK”的形式填入日期， 请尽可能填入完整的日期。
4. CRF 中需填入数值的部位均预留了空格，如“|__|__|__|”，填写时请将个位数字填入最右方的空格，如左侧留有空位，请填入“0”，如：患者血压为 120/80mmHg，则填入“血压：|1|2|0|/ |0|8|0| mmHg”。
5. 请务必完成每页 CRF 最上部分的内容，包括：

① 患者姓名拼音缩写四格需填满，两字姓名填写两字拼音前两个字母；三字姓名填写三字首字母及第三字第二字母；四字姓名填写每一个字首字母。举例：张红 Z H H O 李淑明 L S M I 欧阳小惠 O Y X H 。

② 编号。

③ 访视日期。

课题号	编号	患者姓名缩写	访视日期
	□□□	□□□□	|_|_|_|_|/|_|_|/|_|_| 年 月 日

入组标准： **是 否**

1、年龄为 18 至 70 岁,男女不限； □ □

2、原发病为乙肝相关性肝病或接受单纯核心抗体阳性供肝的受体； □ □

3、肝移植术后至少 12 个月； □ □

4、无乙肝复发迹象，即血清 HBsAg（-）且 HBV DNA（-）； □ □

5、肝功能正常或基本正常（指标升高程度不超过正常值 2 倍）； □ □

6、接种时无排斥反应发生，无近期（1 月内）感染； □ □

7、处于轻度免疫抑制状态，即服用单种免疫抑制剂，或小剂量 □ □

联用两种或多种免疫抑制剂，并停用激素至少半年。

8、充分理解实验研究过程并签署知情同意书。 □ □

如以上任何一个答案为“否”，此受试者不能参加试验

排除标准： **是 否**

1、患者出现肿瘤复发； □ □

2、出现胆道并发症：胆道狭窄、胆漏、胆泥形成、缺血性改变； □ □

3、出现血管并发症：肝动脉、门静脉或下腔静脉出现狭窄、出血、 □ □

血栓形成；

4、合并 HCV、HDV、HIV 感染； □ □

5、合并肾功能不全，需要透析治疗； □ □

6、明确器质性心脏病或心律失常者； □ □

7、妊娠试验阳性或哺乳期妇女，计划近期内生育的男女； □ □

8、既往明确对疫苗任何成分存在超敏反应者。 □ □

如以上任何一个答案为“是”，此受试者不能参加试验

研究基本流程

1、肝移植术后符合入组条件的患者在签署知情同意书后，于 0、1、2、6、12 月各接种一次乙肝疫苗，并于接种完最后一针后至少随访 6 月。

2、患者于每次接种前，常规复查血常规、出凝血常规、肝酶学、肝代谢、基础代谢生化、药物浓度、乙肝两对半定量、乙型肝炎病毒 DNA 定量、肝胆胰脾 B 超并记录近期免疫抑制剂及 HBIG 的使用方案、并发症及不良反应等。

3、接种疫苗期间（接种第一针至接种第五针期间），随访计划在不影响疫苗接种的情况下，可根据患者病情、HBsAb 抗体滴度及患者家庭情况等，经患者与主管医生共同协商后制定。

4、研究随访期（接种第五针至其后随访半年），应停用 HBIG，并尽量每月复查一次乙肝两对半定量、乙型肝炎病毒 DNA 定量，并于随访第 6 月常规复查血常规、出凝血常规、肝酶学、肝代谢、基础代谢生化、药物浓度、乙肝两对半定量、乙型肝炎病毒 DNA 定量、肝胆胰脾 B 超。

5、研究随访期,若患者出现 HBsAb 抗体滴度低于 10 IU/L,将退出该研究，

并重新使用 HBIG 维持 HBsAb 抗体大于 30 IU/L 抗病毒。

课题号	编号	患者姓名缩写	访视日期
	□□□	□□□□	|_|_|_|_|/|_|_|/|_|_| 年 月 日

一般资料及术前情况

性别：男□ 女□ 血型：|_|_|型 身高：|_|.|_|_|m 体重：|_|_|.|_|Kg

联系电话：|_|_|_|_|_|_|_|_|_|_|_| 出生日期：|_|_|_|_|/|_|_|/|_|_|

吸烟：有□ 无□ 平均|_|_|支/日 吸烟年限：|_|_|年

饮酒：有□ 无□ 平均|_|_|_|_|mL/日 饮酒年限：|_|_|年

原发病诊断（尽量详细）：____________________________

____

肝性脑病：无□ 1-2 级□ 3-4 级□ 腹水：无□ 轻度□ 中重度□

ALB：|_|_|.|_|g/L TBil：|_|_|_|.|_|umol/L CR：|_|_|_|umol/L

INR：|_|.|_|_| PT：|_|_|.|_|s

HBsAg：|_|_|_|_|.|_|_|IU/mL HBsAb：|_|_|_|_|.|_|_|IU/L

HBeAg：|_|_|_|_|.|_|_|S/CO HBeAb：|_|_|_|_|.|_|_|S/CO

HBcAb：|_|_|_|_|.|_|_|S/CO HBV-DNA：|_|.|_|_|×10^|_|_|IU/mL

肝移植手术日期：|_|_|_|_|/|_|_|/|_|_|

重要病史摘要

____

____

-

供体情况

HBsAg：|_|_|_|_|.|_|_|IU/mL HBsAb：|_|_|_|_|.|_|_|IU/L

HBeAg：|_|_|_|_|.|_|_|S/CO HBeAb：|_|_|_|_|.|_|_|S/CO

HBcAb：|_|_|_|_|.|_|_|S/CO HCV-Ab：阴□ 阳□

供肝来源：活体□ DCD□ DBD□ 尸体□ 全肝移植：是□ 否□

观察医生：________ 记录日期： ___年_月_日

课题号	访视 1（0月）<br>接种第一针	编号<br>□□□	患者姓名缩写<br>□□□□	访视日期<br>|_|_|_|_|/|_|_|/|_|_| 年 月 日

一般情况：

身高：|_|.|_|_|m 体重：|_|_|.|_|Kg

吸烟：有□ 无□ 平均|_|_|支/日 吸烟年限：|_|_|年

饮酒：有□ 无□ 平均|_|_|_|_|mL/日 饮酒年限：|_|_|年

血常规：

WBC：|_|_|.|_|_|×10^{9}/L 中性粒细胞比例：0.|_|_|_| 淋巴细胞比例：0.|_|_|_|

单核细胞比例：0.|_|_|_| 嗜酸性粒细胞比例：0.|_|_|_|

嗜碱性粒细胞比例：0.|_|_|_| Hb：|_|_|_|g/L PLT: |_|_|_|×10^{9}/L

乙肝两对半定量及 DNA：

HBsAg：|_|_|_|_|.|_|_|IU/mL HBsAb：|_|_|_|_|.|_|_|IU/L

HBeAg：|_|_|_|_|.|_|_|S/CO HBeAb：|_|_|_|_|.|_|_|S/CO

HBcAb：|_|_|_|_|.|_|_|S/CO HBV-DNA：|_|.|_|_|×10^|_|_|IU/mL

肝功能：

ALB：|_|_|.|_|g/L GLB：|_|_|.|_|g/L TBil：|_|_|_|.|_|umol/L

ALT：|_|_|_|IU/L AST：|_|_|_|IU/L ALP：|_|_|_|U/L γ-GGT：|_|_|_|U/L

肾功能：

CR：|_|_|_|umol/L UA：|_|_|_|umol/L UREA：|_|_|.|_|mmol/L

凝血功能：

PT：|_|_|.|_|s APTT：|_|_|.|_|s INR：|_|.|_|_| Fbg：|_|.|_|_|g/L

免疫抑制情况：

他克莫司用量：|_|.|_|mg 频次：QD□ Q12H□ FK506 浓度：|_|_|.|_|_|ug/L

西罗莫司用量：|_|.|_|mg 频次：QD□ Q12H□ 雷帕霉素浓度：|_|_|.|_|_|ng/mL

环孢素用量：|_|_|_|mg 频次：QD□ Q12H□ CsA 浓度：|_|_|_|.|_|_|ug/L

吗替麦考酚酯用量：|_|.|_|_|g 频次：QD□ Q12H□ MMF 浓度：|_|_|.|_|_

|ug/mL

抗乙肝病毒药物使用情况：

药物名称：______ 剂量：|_|_|_|.|_|mg 频次：QD□ Q12H□

HBIG 使用情况：

上次使用时间：|_|_|_|_|/|_|_|/|_|_| 使用剂量：|_|_|_|_|U

用法：im□ iv drip□

腹部彩超：

肝脏质地：______ 血管情况：______

胆道情况：______ 脾脏大小：______

并发症：

肿瘤复发：有□ 无□ 感染：________ 其他：_________

疫苗接种情况：

不良反应：_________________ 退出：是□ 否□

观察医生：__________ 记录日期： ___年_月_日

课题号	访视 2（1 月）<br>接种第二针	编号<br>□□□	患者姓名缩写<br>□□□□	访视日期<br>|_|_|_|_|/|_|_|/|_|_| 年 月 日

一般情况：

身高：|_|.|_|_|m 体重：|_|_|.|_|Kg

吸烟：有□ 无□ 平均|_|_|支/日 吸烟年限：|_|_|年

饮酒：有□ 无□ 平均|_|_|_|_|mL/日 饮酒年限：|_|_|年

血常规：

WBC：|_|_|.|_|_|×10^9/L 中性粒细胞比例：0.|_|_|_| 淋巴细胞比例：0.|_|_|_|

单核细胞比例：0.|_|_|_| 嗜酸性粒细胞比例：0.|_|_|_|

嗜碱性粒细胞比例：0.|_|_|_| Hb：|_|_|_|g/L PLT: |_|_|_|×10^9/L

乙肝两对半定量及 DNA：

HBsAg：|_|_|_|_|.|_|_|IU/mL HBsAb：|_|_|_|_|.|_|_|IU/L
HBeAg：|_|_|_|_|.|_|_|S/CO HBeAb：|_|_|_|_|.|_|_|S/CO
HBcAb：|_|_|_|_|.|_|_|S/CO HBV-DNA：|_|.|_|_|×10^|_|_|IU/mL

肝功能：

ALB：|_|_|.|_|g/L GLB：|_|_|.|_|g/L TBil：|_|_|_|.|_|umol/L
ALT：|_|_|_|IU/L AST：|_|_|_|IU/L ALP：|_|_|_|U/L γ-GGT：|_|_|_|U/L

肾功能：

CR：|_|_|_|umol/L UA：|_|_|_|umol/L UREA：|_|_|.|_|mmol/L

凝血功能：

PT：|_|_|.|_|s APTT：|_|_|.|_|s INR：|_|.|_|_| Fbg：|_|.|_|_|g/L

免疫抑制情况：

他克莫司用量：|_|.|_|mg 频次：QD□ Q12H□ FK506 浓度：|_|_|.|_|_|ug/L

西罗莫司用量：|_|.|_|mg 频次：QD□ Q12H□ 雷帕霉素浓度：|_|_|.|_|_|ng/mL

环孢素用量：|_|_|_|mg 频次：QD□ Q12H□ CsA 浓度：|_|_|_|.|_|_|ug/L

吗替麦考酚酯用量：|_|.|_|_|g 频次：QD□ Q12H□ MMF 浓度：|_|_|.|_|_

|ug/mL

抗乙肝病毒药物使用情况：

药物名称：______ 剂量：|_|_|_|.|_|mg 频次：QD□ Q12H□

HBIG 使用情况：

上次使用时间：|_|_|_|_|/|_|_|/|_|_| 使用剂量：|_|_|_|_|U

用法：im□ iv drip□

腹部彩超：

肝脏质地：______ 血管情况：______
胆道情况：______ 脾脏大小：______

并发症：

肿瘤复发：有□ 无□ 感染：________ 其他：_________

疫苗接种情况：

不良反应：_________________ 退出：是□ 否□

观察医生：__________ 记录日期： ___年_月_日

课题号	访视3（2月）<br>接种第三针	编号<br>□□□	患者姓名缩写<br>□□□□	访视日期<br>|_|_|_|_|/|_|_|/|_|_| 年 月 日

一般情况：

身高：|_|.|_|_|m 体重：|_|_|.|_|Kg

吸烟：有□ 无□ 平均|_|_|支/日 吸烟年限：|_|_|年

饮酒：有□ 无□ 平均|_|_|_|_|mL/日 饮酒年限：|_|_|年

血常规：

WBC：|_|_|.|_|_|×10^{9}/L 中性粒细胞比例：0.|_|_|_| 淋巴细胞比例：0.|_|_|_|

单核细胞比例：0.|_|_|_| 嗜酸性粒细胞比例：0.|_|_|_|

嗜碱性粒细胞比例：0.|_|_|_| Hb：|_|_|_|g/L PLT: |_|_|_|×10^{9}/L

乙肝两对半定量及DNA：

HBsAg：|_|_|_|_|.|_|_|IU/mL HBsAb：|_|_|_|_|.|_|_|IU/L
HBeAg：|_|_|_|_|.|_|_|S/CO HBeAb：|_|_|_|_|.|_|_|S/CO
HBcAb：|_|_|_|_|.|_|_|S/CO HBV-DNA：|_|.|_|_|×10^|_|_|IU/mL

肝功能：

ALB：|_|_|.|_|g/L GLB：|_|_|.|_|g/L TBil：|_|_|_|.|_|umol/L
ALT：|_|_|_|IU/L AST：|_|_|_|IU/L ALP：|_|_|_|U/L γ-GGT：|_|_|_|U/L

肾功能：

CR：|_|_|_|umol/L UA：|_|_|_|umol/L UREA：|_|_|.|_|mmol/L

凝血功能：

PT：|_|_|.|_|s APTT：|_|_|.|_|s INR：|_|.|_|_| Fbg：|_|.|_|_|g/L

免疫抑制情况：

他克莫司用量：|_|.|_|mg 频次：QD□ Q12H□ FK506浓度：|_|_|.|_|_|ug/L

西罗莫司用量：|_|.|_|mg 频次：QD□ Q12H□ 雷帕霉素浓度：|_|_|.|_|_|ng/mL

环孢素用量：|_|_|_|mg 频次：QD□ Q12H□ CsA浓度：|_|_|_|.|_|_|ug/L

吗替麦考酚酯用量：|_|.|_|_|g 频次：QD□ Q12H□ MMF浓度：|_|_|.|_|_

|ug/mL

抗乙肝病毒药物使用情况：

药物名称：______ 剂量：|_|_|_|.|_|mg 频次：QD□ Q12H□

HBIG 使用情况：

上次使用时间：|_|_|_|_|/|_|_|/|_|_| 使用剂量：|_|_|_|_|U

用法：im□ iv drip□

腹部彩超：

肝脏质地：______ 血管情况：______

胆道情况：______ 脾脏大小：______

并发症：

肿瘤复发：有□ 无□ 感染：________ 其他：_________

疫苗接种情况：

不良反应：_________________ 退出：是□ 否□

观察医生：__________ 记录日期： ___年_月_日

课题号	访视4（6月）<br>接种第四针	编号<br>□□□	患者姓名缩写<br>□□□□	访视日期<br>|_|_|_|_|/|_|_|/|_|_| 年 月 日

一般情况：

身高：|_|.|_|_|m 体重：|_|_|.|_|Kg

吸烟：有□ 无□ 平均|_|_|支/日 吸烟年限：|_|_|年

饮酒：有□ 无□ 平均|_|_|_|_|mL/日 饮酒年限：|_|_|年

血常规：

WBC：|_|_|.|_|_|×10^{9}/L 中性粒细胞比例：0.|_|_|_| 淋巴细胞比例：0.|_|_|_|

单核细胞比例：0.|_|_|_| 嗜酸性粒细胞比例：0.|_|_|_|

嗜碱性粒细胞比例：0.|_|_|_| Hb：|_|_|_|g/L PLT: |_|_|_|×10^{9}/L

乙肝两对半定量及DNA：

HBsAg：|_|_|_|_|.|_|_|IU/mL HBsAb：|_|_|_|_|.|_|_|IU/L
HBeAg：|_|_|_|_|.|_|_|S/CO HBeAb：|_|_|_|_|.|_|_|S/CO
HBcAb：|_|_|_|_|.|_|_|S/CO HBV-DNA：|_|.|_|_|×10^|_|_|IU/mL

肝功能：

ALB：|_|_|.|_|g/L GLB：|_|_|.|_|g/L TBil：|_|_|_|.|_|umol/L
ALT：|_|_|_|IU/L AST：|_|_|_|IU/L ALP：|_|_|_|U/L γ-GGT：|_|_|_|U/L

肾功能：

CR：|_|_|_|umol/L UA：|_|_|_|umol/L UREA：|_|_|.|_|mmol/L

凝血功能：

PT：|_|_|.|_|s APTT：|_|_|.|_|s INR：|_|.|_|_| Fbg：|_|.|_|_|g/L

免疫抑制情况：

他克莫司用量：|_|.|_|mg 频次：QD□ Q12H□ FK506浓度：|_|_|.|_|_|ug/L

西罗莫司用量：|_|.|_|mg 频次：QD□ Q12H□ 雷帕霉素浓度：|_|_|.|_|_|ng/mL

环孢素用量：|_|_|_|mg 频次：QD□ Q12H□ CsA浓度：|_|_|_|.|_|_|ug/L

吗替麦考酚酯用量：|_|.|_|_|g 频次：QD□ Q12H□ MMF浓度：|_|_|.|_|_

|ug/mL

抗乙肝病毒药物使用情况：

药物名称：______ 剂量：|_|_|_|.|_|mg 频次：QD□ Q12H□

HBIG 使用情况：

上次使用时间：|_|_|_|_|/|_|_|/|_|_| 使用剂量：|_|_|_|_|U

用法：im□ iv drip□

腹部彩超：

肝脏质地：______ 血管情况：______

胆道情况：______ 脾脏大小：______

并发症：

肿瘤复发：有□ 无□ 感染：________ 其他：_________

疫苗接种情况：

不良反应：_________________ 退出：是□ 否□

观察医生：__________ 记录日期： ___年_月_日

课题号	访视 5（12月）<br><br>接种第五针	编号<br><br>□□□	患者姓名缩写<br><br>□□□□	访视日期<br><br>|_|_|_|_|/|_|_|/|_|_| <br>年　月　日

一般情况：

身高：|_|.|_|_|m 体重：|_|_|.|_|Kg

吸烟：有□ 无□ 平均|_|_|支/日 吸烟年限：|_|_|年

饮酒：有□ 无□ 平均|_|_|_|_|mL/日 饮酒年限：|_|_|年

血常规：

WBC：|_|_|.|_|_|×10^9/L 中性粒细胞比例：0.|_|_|_| 淋巴细胞比例：0.|_|_|_|

单核细胞比例：0.|_|_|_| 嗜酸性粒细胞比例：0.|_|_|_|

嗜碱性粒细胞比例：0.|_|_|_| Hb：|_|_|_|g/L PLT: |_|_|_|×10^9/L

乙肝两对半定量及 DNA：

HBsAg：|_|_|_|_|.|_|_|IU/mL HBsAb：|_|_|_|_|.|_|_|IU/L
HBeAg：|_|_|_|_|.|_|_|S/CO HBeAb：|_|_|_|_|.|_|_|S/CO
HBcAb：|_|_|_|_|.|_|_|S/CO HBV-DNA：|_|.|_|_|×10^|_|_|IU/mL

肝功能：

ALB：|_|_|.|_|g/L GLB：|_|_|.|_|g/L TBil：|_|_|_|.|_|umol/L
ALT：|_|_|_|IU/L AST：|_|_|_|IU/L ALP：|_|_|_|U/L γ-GGT：|_|_|_|U/L

肾功能：

CR：|_|_|_|umol/L UA：|_|_|_|umol/L UREA：|_|_|.|_|mmol/L

凝血功能：

PT：|_|_|.|_|s APTT：|_|_|.|_|s INR：|_|.|_|_| Fbg：|_|.|_|_|g/L

免疫抑制情况：

他克莫司用量：|_|.|_|mg 频次：QD□ Q12H□ FK506 浓度：|_|_|.|_|_|ug/L

西罗莫司用量：|_|.|_|mg 频次：QD□ Q12H□ 雷帕霉素浓度：|_|_|.|_|_|ng/mL

环孢素用量：|_|_|_|mg 频次：QD□ Q12H□ CsA 浓度：|_|_|_|.|_|_|ug/L

吗替麦考酚酯用量：|_|.|_|_|g 频次：QD□ Q12H□ MMF 浓度：|_|_|.|_|_|ug/mL

抗乙肝病毒药物使用情况：

药物名称：______ 剂量：|_|_|_|.|_|mg 频次：QD□ Q12H□

HBIG 使用情况：

上次使用时间：|_|_|_|_|/|_|_|/|_|_| 使用剂量：|_|_|_|_|U

用法：im□ iv drip□

腹部彩超：

肝脏质地：_______ 血管情况：_______

胆道情况：_______ 脾脏大小：_______

并发症：

肿瘤复发：有□ 无□ 感染：_________ 其他：_________

疫苗接种情况：

不良反应：__________________ 退出：是□ 否□

观察医生：__________ 记录日期： ___年_月_日

课题号	访视 6（13 月）	编号<br><br>□□□	患者姓名缩写<br><br>□□□□	访视日期<br>|_|_|_|_|/|_|_|/|_|_| 年 月 日

一般情况：

身高：|_|.|_|_|m 体重：|_|_|.|_|Kg

吸烟：有□ 无□ 平均|_|_|支/日 吸烟年限：|_|_|年

饮酒：有□ 无□ 平均|_|_|_|_|mL/日 饮酒年限：|_|_|年

血常规：

WBC：|_|_|.|_|_|×10^9/L 中性粒细胞比例：0.|_|_|_| 淋巴细胞比例：0.|_|_|_|

单核细胞比例：0.|_|_|_| 嗜酸性粒细胞比例：0.|_|_|_|

嗜碱性粒细胞比例：0.|_|_|_| Hb：|_|_|_|g/L PLT: |_|_|_|×10^9/L

乙肝两对半定量及 DNA：

HBsAg：|_|_|_|_|.|_|_|IU/mL HBsAb：|_|_|_|_|.|_|_|IU/L
HBeAg：|_|_|_|_|.|_|_|S/CO HBeAb：|_|_|_|_|.|_|_|S/CO
HBcAb：|_|_|_|_|.|_|_|S/CO HBV-DNA：|_|.|_|_|×10^|_|_|IU/mL

肝功能：

ALB：|_|_|.|_|g/L GLB：|_|_|.|_|g/L TBil：|_|_|_|.|_|umol/L
ALT：|_|_|_|IU/L AST：|_|_|_|IU/L ALP：|_|_|_|U/L γ-GGT：|_|_|_|U/L

肾功能：

CR：|_|_|_|umol/L UA：|_|_|_|umol/L UREA：|_|_|.|_|mmol/L

凝血功能：

PT：|_|_|.|_|s APTT：|_|_|.|_|s INR：|_|.|_|_| Fbg：|_|.|_|_|g/L

免疫抑制情况：

他克莫司用量：|_|.|_|mg 频次：QD□ Q12H□ FK506 浓度：|_|_|.|_|_|ug/L

西罗莫司用量：|_|.|_|mg 频次：QD□ Q12H□ 雷帕霉素浓度：|_|_|.|_|_|ng/mL

环孢素用量：|_|_|_|mg 频次：QD□ Q12H□ CsA 浓度：|_|_|_|.|_|_|ug/L

吗替麦考酚酯用量：|_|.|_|_|g 频次：QD□ Q12H□ MMF 浓度：|_|_|.|_|_|ug/mL

抗乙肝病毒药物使用情况：

药物名称：______ 剂量：|_|_|_|.|_|mg 频次：QD□ Q12H□

HBIG 使用情况：

上次使用时间：|_|_|_|_|/|_|_|/|_|_| 使用剂量：|_|_|_|_|U

用法：im□ iv drip□

腹部彩超：

肝脏质地：_______ 血管情况：_______

胆道情况：_______ 脾脏大小：_______

并发症：

肿瘤复发：有□ 无□ 感染：_________ 其他：_________

疫苗接种情况：

不良反应：__________________ 退出：是□ 否□

观察医生：__________　　　记录日期：___年_月_日

课题号	访视 7（14 月）	编号<br><br>□□□	患者姓名缩写<br><br>□□□□	访视日期<br>|_|_|_|_|/|_|_|/|_|_| 年 月 日

一般情况：

身高：|_|.|_|_|m 体重：|_|_|.|_|Kg

吸烟：有□ 无□ 平均|_|_|支/日 吸烟年限：|_|_|年

饮酒：有□ 无□ 平均|_|_|_|_|mL/日 饮酒年限：|_|_|年

血常规：

WBC：|_|_|.|_|_|×10^9/L 中性粒细胞比例：0.|_|_|_| 淋巴细胞比例：0.|_|_|_|

单核细胞比例：0.|_|_|_| 嗜酸性粒细胞比例：0.|_|_|_|

嗜碱性粒细胞比例：0.|_|_|_| Hb：|_|_|_|g/L PLT: |_|_|_|×10^9/L

乙肝两对半定量及 DNA：

HBsAg：|_|_|_|_|.|_|_|IU/mL HBsAb：|_|_|_|_|.|_|_|IU/L
HBeAg：|_|_|_|_|.|_|_|S/CO HBeAb：|_|_|_|_|.|_|_|S/CO
HBcAb：|_|_|_|_|.|_|_|S/CO HBV-DNA：|_|.|_|_|×10^|_|_|IU/mL

肝功能：

ALB：|_|_|.|_|g/L GLB：|_|_|.|_|g/L TBil：|_|_|_|.|_|umol/L
ALT：|_|_|_|IU/L AST：|_|_|_|IU/L ALP：|_|_|_|U/L γ-GGT：|_|_|_|U/L

肾功能：

CR：|_|_|_|umol/L UA：|_|_|_|umol/L UREA：|_|_|.|_|mmol/L

凝血功能：

PT：|_|_|.|_|s APTT：|_|_|.|_|s INR：|_|.|_|_| Fbg：|_|.|_|_|g/L

免疫抑制情况：

他克莫司用量：|_|.|_|mg 频次：QD□ Q12H□ FK506 浓度：|_|_|.|_|_|ug/L

西罗莫司用量：|_|.|_|mg 频次：QD□ Q12H□ 雷帕霉素浓度：|_|_|.|_|_|ng/mL

环孢素用量：|_|_|_|mg 频次：QD□ Q12H□ CsA 浓度：|_|_|_|.|_|_|ug/L

吗替麦考酚酯用量：|_|.|_|_|g 频次：QD□ Q12H□ MMF 浓度：|_|_|.|_|_|ug/mL

抗乙肝病毒药物使用情况：

药物名称：______ 剂量：|_|_|_|.|_|mg 频次：QD□ Q12H□

HBIG 使用情况：

上次使用时间：|_|_|_|_|/|_|_|/|_|_| 使用剂量：|_|_|_|_|U

用法：im□ iv drip□

腹部彩超：

肝脏质地：_______ 血管情况：_______

胆道情况：_______ 脾脏大小：_______

并发症：

肿瘤复发：有□ 无□ 感染：_________ 其他：_________

疫苗接种情况：

不良反应：__________________ 退出：是□ 否□

观察医生：________ 记录日期： ___年_月_日

课题号	访视 8（15月）	编号<br><br>□□□	患者姓名缩写<br><br>□□□□	访视日期 |_|_|_|_|/|_|_|/|_|_| <br>年 月 日

一般情况：

身高：|_|.|_|_|m 体重：|_|_|.|_|Kg

吸烟：有□ 无□ 平均|_|_|支/日 吸烟年限：|_|_|年

饮酒：有□ 无□ 平均|_|_|_|_|mL/日 饮酒年限：|_|_|年

血常规：

WBC：|_|_|.|_|_|×10^9/L 中性粒细胞比例：0.|_|_|_| 淋巴细胞比例：0.|_|_|_|

单核细胞比例：0.|_|_|_| 嗜酸性粒细胞比例：0.|_|_|_|

嗜碱性粒细胞比例：0.|_|_|_| Hb：|_|_|_|g/L PLT: |_|_|_|×10^9/L

乙肝两对半定量及 DNA：

HBsAg：|_|_|_|_|.|_|_|IU/mL HBsAb：|_|_|_|_|.|_|_|IU/L
HBeAg：|_|_|_|_|.|_|_|S/CO HBeAb：|_|_|_|_|.|_|_|S/CO
HBcAb：|_|_|_|_|.|_|_|S/CO HBV-DNA：|_|.|_|_|×10^|_|_|IU/mL

肝功能：

ALB：|_|_|.|_|g/L GLB：|_|_|.|_|g/L TBil：|_|_|_|.|_|umol/L
ALT：|_|_|_|IU/L AST：|_|_|_|IU/L ALP：|_|_|_|U/L γ-GGT：|_|_|_|U/L

肾功能：

CR：|_|_|_|umol/L UA：|_|_|_|umol/L UREA：|_|_|.|_|mmol/L

凝血功能：

PT：|_|_|.|_|s APTT：|_|_|.|_|s INR：|_|.|_|_| Fbg：|_|.|_|_|g/L

免疫抑制情况：

他克莫司用量：|_|.|_|mg 频次：QD□ Q12H□ FK506 浓度：|_|_|.|_|_|ug/L

西罗莫司用量：|_|.|_|mg 频次：QD□ Q12H□ 雷帕霉素浓度：|_|_|.|_|_|ng/mL

环孢素用量：|_|_|_|mg 频次：QD□ Q12H□ CsA 浓度：|_|_|_|.|_|_|ug/L

吗替麦考酚酯用量：|_|.|_|_|g 频次：QD□ Q12H□ MMF 浓度：|_|_|.|_|_|ug/mL

抗乙肝病毒药物使用情况：

药物名称：______ 剂量：|_|_|_|.|_|mg 频次：QD□ Q12H□

HBIG 使用情况：

上次使用时间：|_|_|_|_|/|_|_|/|_|_| 使用剂量：|_|_|_|_|U

用法：im□ iv drip□

腹部彩超：

肝脏质地：_______ 血管情况：_______

胆道情况：_______ 脾脏大小：_______

并发症：

肿瘤复发：有□ 无□ 感染：_________ 其他：_________

疫苗接种情况：

不良反应：__________________ 退出：是□ 否□

观察医生：__________　　　记录日期：___年_月_日

课题号	访视 9（16 月）	编号<br><br>□□□	患者姓名缩写<br><br>□□□□	访视日期 |_|_|_|_|/|_|_|/|_|_| 年 月 日

一般情况：

身高：|_|.|_|_|m 体重：|_|_|.|_|Kg

吸烟：有□ 无□ 平均|_|_|支/日 吸烟年限：|_|_|年

饮酒：有□ 无□ 平均|_|_|_|_|mL/日 饮酒年限：|_|_|年

血常规：

WBC：|_|_|.|_|_|×10^9/L 中性粒细胞比例：0.|_|_|_| 淋巴细胞比例：0.|_|_|_|

单核细胞比例：0.|_|_|_| 嗜酸性粒细胞比例：0.|_|_|_|

嗜碱性粒细胞比例：0.|_|_|_| Hb：|_|_|_|g/L PLT: |_|_|_|×10^9/L

乙肝两对半定量及 DNA：

HBsAg：|_|_|_|_|.|_|_|IU/mL HBsAb：|_|_|_|_|.|_|_|IU/L
HBeAg：|_|_|_|_|.|_|_|S/CO HBeAb：|_|_|_|_|.|_|_|S/CO
HBcAb：|_|_|_|_|.|_|_|S/CO HBV-DNA：|_|.|_|_|×10^|_|_|IU/mL

肝功能：

ALB：|_|_|.|_|g/L GLB：|_|_|.|_|g/L TBil：|_|_|_|.|_|umol/L
ALT：|_|_|_|IU/L AST：|_|_|_|IU/L ALP：|_|_|_|U/L γ-GGT：|_|_|_|U/L

肾功能：

CR：|_|_|_|umol/L UA：|_|_|_|umol/L UREA：|_|_|.|_|mmol/L

凝血功能：

PT：|_|_|.|_|s APTT：|_|_|.|_|s INR：|_|.|_|_| Fbg：|_|.|_|_|g/L

免疫抑制情况：

他克莫司用量：|_|.|_|mg 频次：QD□ Q12H□ FK506 浓度：|_|_|.|_|_|ug/L

西罗莫司用量：|_|.|_|mg 频次：QD□ Q12H□ 雷帕霉素浓度：|_|_|.|_|_|ng/mL

环孢素用量：|_|_|_|mg 频次：QD□ Q12H□ CsA 浓度：|_|_|_|.|_|_|ug/L

吗替麦考酚酯用量：|_|.|_|_|g 频次：QD□ Q12H□ MMF 浓度：|_|_|.|_|_|ug/mL

抗乙肝病毒药物使用情况：

药物名称：______ 剂量：|_|_|_|.|_|mg 频次：QD□ Q12H□

HBIG 使用情况：

上次使用时间：|_|_|_|_|/|_|_|/|_|_| 使用剂量：|_|_|_|_|U

用法：im□ iv drip□

腹部彩超：

肝脏质地：______ 血管情况：______
胆道情况：______ 脾脏大小：______

并发症：

肿瘤复发：有□ 无□ 感染：________ 其他：_________

疫苗接种情况：

不良反应：_________________ 退出：是□ 否□

观察医生：__________　　记录日期：___年_月_日

课题号	访视 10（17 月）	编号	患者姓名缩写	访视日期
		□□□	□□□□	|_|_|_|_|/|_|_|/|_|_| 年 月 日

一般情况：

身高：|_|.|_|_|m 体重：|_|_|.|_|Kg

吸烟：有□ 无□ 平均|_|_|支/日 吸烟年限：|_|_|年

饮酒：有□ 无□ 平均|_|_|_|_|mL/日 饮酒年限：|_|_|年

血常规：

WBC：|_|_|.|_|_|×10^9/L 中性粒细胞比例：0.|_|_|_| 淋巴细胞比例：0.|_|_|_|

单核细胞比例：0.|_|_|_| 嗜酸性粒细胞比例：0.|_|_|_|

嗜碱性粒细胞比例：0.|_|_|_| Hb：|_|_|_|g/L PLT: |_|_|_|×10^9/L

乙肝两对半定量及 DNA：

HBsAg：|_|_|_|_|.|_|_|IU/mL HBsAb：|_|_|_|_|.|_|_|IU/L
HBeAg：|_|_|_|_|.|_|_|S/CO HBeAb：|_|_|_|_|.|_|_|S/CO
HBcAb：|_|_|_|_|.|_|_|S/CO HBV-DNA：|_|.|_|_|×10^|_|_|IU/mL

肝功能：
ALB：|_|_|.|_|g/L GLB：|_|_|.|_|g/L TBil：|_|_|_|.|_|umol/L
ALT：|_|_|_|IU/L AST：|_|_|_|IU/L ALP：|_|_|_|U/L γ-GGT：|_|_|_|U/L
肾功能：
CR：|_|_|_|umol/L UA：|_|_|_|umol/L UREA：|_|_|.|_|mmol/L
凝血功能：
PT：|_|_|.|_|s APTT：|_|_|.|_|s INR：|_|.|_|_| Fbg：|_|.|_|_|g/L
免疫抑制情况：

他克莫司用量：|_|.|_|mg 频次：QD□ Q12H□ FK506 浓度：|_|_|.|_|_|ug/L

西罗莫司用量：|_|.|_|mg 频次：QD□ Q12H□ 雷帕霉素浓度：|_|_|.|_|_|ng/mL

环孢素用量：|_|_|_|mg 频次：QD□ Q12H□ CsA 浓度：|_|_|_|.|_|_|ug/L

吗替麦考酚酯用量：|_|.|_|_|g 频次：QD□ Q12H□ MMF 浓度：|_|_|.|_|_

|ug/mL

抗乙肝病毒药物使用情况：

药物名称：______ 剂量：|_|_|_|.|_|mg 频次：QD□ Q12H□

HBIG 使用情况：

上次使用时间：|_|_|_|_|/|_|_|/|_|_| 使用剂量：|_|_|_|_|U

用法：im□ iv drip□

腹部彩超：
肝脏质地：______ 血管情况：______
胆道情况：______ 脾脏大小：______
并发症：

肿瘤复发：有□ 无□ 感染：________ 其他：________

疫苗接种情况：

不良反应：________________ 退出：是□ 否□

观察医生：________ 记录日期： ___年_月_日

课题号	访视 11（18月）	编号<br><br>□□□	患者姓名缩写<br><br>□□□□	访视日期<br><br>|_|_|_|_|/|_|_|/|_|_| <br>年 月 日

一般情况：

身高：|_|.|_|_|m 体重：|_|_|.|_|Kg

吸烟：有□ 无□ 平均|_|_|支/日 吸烟年限：|_|_|年

饮酒：有□ 无□ 平均|_|_|_|_|mL/日 饮酒年限：|_|_|年

血常规：

WBC：|_|_|.|_|_|×10⁹/L 中性粒细胞比例：0.|_|_|_| 淋巴细胞比例：0.|_|_|_|

单核细胞比例：0.|_|_|_| 嗜酸性粒细胞比例：0.|_|_|_|

嗜碱性粒细胞比例：0.|_|_|_| Hb：|_|_|_|g/L PLT: |_|_|_|×10⁹/L

乙肝两对半定量及 DNA：

HBsAg：|_|_|_|_|.|_|_|IU/mL HBsAb：|_|_|_|_|.|_|_|IU/L
HBeAg：|_|_|_|_|.|_|_|S/CO HBeAb：|_|_|_|_|.|_|_|S/CO
HBcAb：|_|_|_|_|.|_|_|S/CO HBV-DNA：|_|.|_|_|×10^|_|_|IU/mL

肝功能：

ALB：|_|_|.|_|g/L GLB：|_|_|.|_|g/L TBil：|_|_|_|.|_|umol/L
ALT：|_|_|_|IU/L AST：|_|_|_|IU/L ALP：|_|_|_|U/L γ-GGT：|_|_|_|U/L

肾功能：

CR：|_|_|_|umol/L UA：|_|_|_|umol/L UREA：|_|_|.|_|mmol/L

凝血功能：

PT：|_|_|.|_|s APTT：|_|_|.|_|s INR：|_|.|_|_| Fbg：|_|.|_|_|g/L

免疫抑制情况：

他克莫司用量：|_|.|_|mg 频次：QD□ Q12H□ FK506 浓度：|_|_|.|_|_|ug/L

西罗莫司用量：|_|.|_|mg 频次：QD□ Q12H□ 雷帕霉素浓度：|_|_|.|_|_|ng/mL

环孢素用量：|_|_|_|mg 频次：QD□ Q12H□ CsA 浓度：|_|_|_|.|_|_|ug/L

吗替麦考酚酯用量：|_|.|_|_|g 频次：QD□ Q12H□ MMF 浓度：|_|_|.|_|_|ug/mL

抗乙肝病毒药物使用情况：

药物名称：______ 剂量：|_|_|_|.|_|mg 频次：QD□ Q12H□

HBIG 使用情况：

上次使用时间：|_|_|_|_|/|_|_|/|_|_| 使用剂量：|_|_|_|_|U

用法：im□ iv drip□

腹部彩超：

肝脏质地：_______ 血管情况：_______

胆道情况：_______ 脾脏大小：_______

并发症：

肿瘤复发：有□ 无□ 感染：_________ 其他：_________

疫苗接种情况：

不良反应：__________________ 退出：是□ 否□

观察医生：________　　　记录日期： ___年_月_日

不良事件（ADVERSE EVENT）

<table>
<tr><td colspan="4">（用标准医学术语）记录所有观察到的和用以下问句“自上次检查后，您有何不同的感觉？”直接询问得出的不良事件。尽量使用诊断名称而不使用症状名称。每一栏记录一个不良事件。
如果在试验期间有不良事件发生，请填写下表。无论有无不良事件发生均应在此表下方签名。
有无不良事件发生？ □ 有 □ 无</td></tr>
<tr><td>不良事件名称
（填写字迹要清晰）</td><td></td><td></td><td></td></tr>
<tr><td>开始发生日期和时间</td><td>______年___月___日
___：__（24 小时制）</td><td>______年___月___日
___：__（24 小时制）</td><td>______年___月___日
___：__（24 小时制）</td></tr>
<tr><td>不良事件严重程度</td><td>□轻 □中 □重</td><td>□轻 □中 □重</td><td>□轻 □中 □重</td></tr>
<tr><td>是否采取措施
（如是，请记录伴随用药和伴随治疗记录表）</td><td>□是 □否
____

-----------------------------------</td><td>□是 □否
____

-----------------------------------</td><td>□是 □否
____

-----------------------------------</td></tr>
<tr><td>根据研究者的判断是否符合严重不良事件定义？</td><td>□ 是 □ 否</td><td>□ 是 □ 否</td><td>□ 是 □ 否</td></tr>
</table>

1.导致死亡.<br>2.威胁生命.<br>3.导致住院或延长住院期间.<br>4.导致持续或严重残疾/能力丧失.<br>5.导致先天性异常或出生缺陷.<br>6.重要医学事件（如有可能影响到受试者并有可能需要药物/手术以防止上述结果）	（如是，请立即电话/传真报告中山大学附属第一医院。）<br><br>报告日期：<br>____年__月__日	（如是，请立即电话/传真报告中山大学附属第一医院。）<br><br>报告日期：<br>____年__月__日	（如是，请立即电话/传真报告中山大学附属第一医院。）<br><br>报告日期：<br>____年__月__日
**在不良事件终止或研究结束时填写以下部分**			
**所发生不良事件的结局**	☐ 仍存在 ☐ 已缓解<br>☐ 不知道<br>缓解日期：<br>____年__月__日	☐ 仍存在 ☐ 已缓解<br>☐ 不知道<br>缓解日期：<br>____年__月__日	☐ 仍存在 ☐ 已缓解<br>☐ 不知道<br>缓解日期：<br>____年__月__日
**患者是否因此不良事件而退出试验?**	☐ 是 ☐ 否	☐ 是 ☐ 否	☐ 是 ☐ 否

伴随治疗记录表

伴随治疗药物种类	药物名称	药物剂量	起止时间

注：请填写免疫抑制剂以外的伴随治疗药物名称，如降压药、降糖药、降脂药、抗病毒药等。

**受试者签署知情同意书日期：**<br><br>**试验开始时间：**

**试验结束时间：**<br><br>**受试者是否中止临床试验？　□否；　□是（如是，请填写以下中止试验原因）**
**受试者中止试验日期**<br><br>**中止试验的主要原因是**：<br>□不良事件（已填写不良事件表）<br>□违背试验方案<br>□受试者撤回知情同意书<br>□其它的原因：________________

观察医生：________　　　　记录日期：__年_月_日

监查员声明

**我确认本份病例报告表所填内容完整、准确、真实、可靠。**<br><br>**监查员签名：_______　　　日期:____年 _月_日**

病例报告表审核声明

**我证实由我签名的此病例报告表的各页已由我检查，并确认所有信息是真实、准确的并符合研究方案的要求。**<br><br>**主要研究者签名:_______　　　日期:____年 _月_日**
